# Temporal change in chromatin accessibility predicts regulators of nodulation in *Medicago truncatula*

**DOI:** 10.1186/s12915-022-01450-9

**Published:** 2022-11-09

**Authors:** Sara A. Knaack, Daniel Conde, Sanhita Chakraborty, Kelly M. Balmant, Thomas B. Irving, Lucas Gontijo Silva Maia, Paolo M. Triozzi, Christopher Dervinis, Wendell J. Pereira, Junko Maeda, Henry W. Schmidt, Jean-Michel Ané, Matias Kirst, Sushmita Roy

**Affiliations:** 1 Wisconsin Institute for Discovery, University of Wisconsin, Madison, WI 53715 USA; 2 School of Forest Resources and Conservation, University of Florida, Gainesville, FL 32611 USA; 3 Department of Bacteriology, University of Wisconsin, Madison, WI 53706 USA; 4 Department of Agronomy, University of Wisconsin, Madison, WI 53706 USA; 5 Genetics Institute, University of Florida, Gainesville, FL 32611 USA; 6 Department of Biostatistics and Medical Informatics, University of Wisconsin, Madison, WI 53792 USA; 7 Department of Computer Sciences, University of Wisconsin, Madison, WI 53792 USA

**Keywords:** Nitrogen fixation, Nodulation, Symbiosis, Chromatin accessibility, Transcriptome and chromatin dynamics, Gene regulatory network, *Cis*-regulatory elements, Machine learning, Medicago

## Abstract

**Background:**

Symbiotic associations between bacteria and leguminous plants lead to the formation of root nodules that fix nitrogen needed for sustainable agricultural systems. Symbiosis triggers extensive genome and transcriptome remodeling in the plant, yet an integrated understanding of the extent of chromatin changes and transcriptional networks that functionally regulate gene expression associated with symbiosis remains poorly understood. In particular, analyses of early temporal events driving this symbiosis have only captured correlative relationships between regulators and targets at mRNA level. Here, we characterize changes in transcriptome and chromatin accessibility in the model legume *Medicago truncatula*, in response to rhizobial signals that trigger the formation of root nodules.

**Results:**

We profiled the temporal chromatin accessibility (ATAC-seq) and transcriptome (RNA-seq) dynamics of *M. truncatula* roots treated with bacterial small molecules called lipo-chitooligosaccharides that trigger host symbiotic pathways of nodule development. Using a novel approach, dynamic regulatory module networks, we integrated ATAC-seq and RNA-seq time courses to predict *cis*-regulatory elements and transcription factors that most significantly contribute to transcriptomic changes associated with symbiosis. Regulators involved in auxin (IAA4-5, SHY2), ethylene (EIN3, ERF1), and abscisic acid (ABI5) hormone response, as well as histone and DNA methylation (IBM1), emerged among those most predictive of transcriptome dynamics. RNAi-based knockdown of EIN3 and ERF1 reduced nodule number in *M. truncatula* validating the role of these predicted regulators in symbiosis between legumes and rhizobia.

**Conclusions:**

Our transcriptomic and chromatin accessibility datasets provide a valuable resource to understand the gene regulatory programs controlling the early stages of the dynamic process of symbiosis. The regulators identified provide potential targets for future experimental validation, and the engineering of nodulation in species is unable to establish that symbiosis naturally.

**Supplementary Information:**

The online version contains supplementary material available at 10.1186/s12915-022-01450-9.

## Introduction

Legumes such as *Medicago truncatula* can establish a well-characterized mutualism with nitrogen-fixing rhizobia. Signal exchanges between the host plant and bacteria initiate intracellular infection of host cells, followed by the development and colonization of root nodules [1]. Nodules provide a unique niche for the bacteria and fix nitrogen. Nodulating plants can grow with little to no outside sources of nitrogen and even build soil nitrogen levels for subsequent crops [2]. Hence, understanding symbiotic processes between legumes and rhizobia is extremely valuable for the productivity and sustainability of agricultural systems worldwide.

Symbiosis begins with compatible rhizobia detecting flavonoids and isoflavonoids produced by the legume host [3] and subsequent release of lipo-chitooligosaccharides (LCOs) by the bacteria. The host plant perceives LCOs with LysM domain receptor-like kinases heterodimers, such as Nod factor perception (NFP) and LysM domain receptor-like kinase 3 (LYK3) in *M. truncatula* [4, 5]. LCO perception activates a signaling cascade, involving the plasma membrane-localized LRR-type receptor kinase doesn’t make infections 2/nodulation receptor kinase (MtDMI2/MtNORK), the calcium-regulated calcium channel (MtDMI1), cyclic nucleotide-gated calcium channels, *M. truncatula* calcium ATPase 8 (MtMCA8), and including the components of the nuclear pore complex [6–8]. The cascade results in oscillations of nuclear calcium concentrations, detectable by the nucleus-localized calcium/calmodulin-dependent protein kinase (CCaMK, MtDMI3 in *M. truncatula*) [9]. CCaMK activates the transcription factor (TF) interacting protein of DMI3 (MtIPD3/CYCLOPS). Downstream, other TFs are activated, such as nodulation-signaling pathway 1 and 2 (NSP1 and NSP2), Nodule INception (NIN), ethylene response factor required for nodulation 1, 2, and 3 (ERN1, 2, and 3), and nuclear factor YA-1 and YB-1 (NF-YA1 and NF-YB-1) [10, 11].

The coordinated activity of these TFs triggers transcriptional changes [12] essential for infection of the root hair cells (in *M. truncatula*), nodule organogenesis, and infection of the nodule cortex [10]. These processes require changes in chromatin accessibility [13] on a continuum from closed to open, which are important for cell function [14]. Chromatin reorganization has been shown to regulate a number of processes in plants including photomorphogenesis and flowering [15, 16]. For example, active DNA demethylation by DEMETER (DME) is critical for gene expression reprogramming during nodule differentiation in *M. truncatula* and the acquisition of organ identity [13]. Also, in *M. truncatula*, the gene expression level of nodule-specific cysteine-rich genes (NCR) across root nodule zones are correlated with chromatin accessibility [17].

**Fig. 1:**
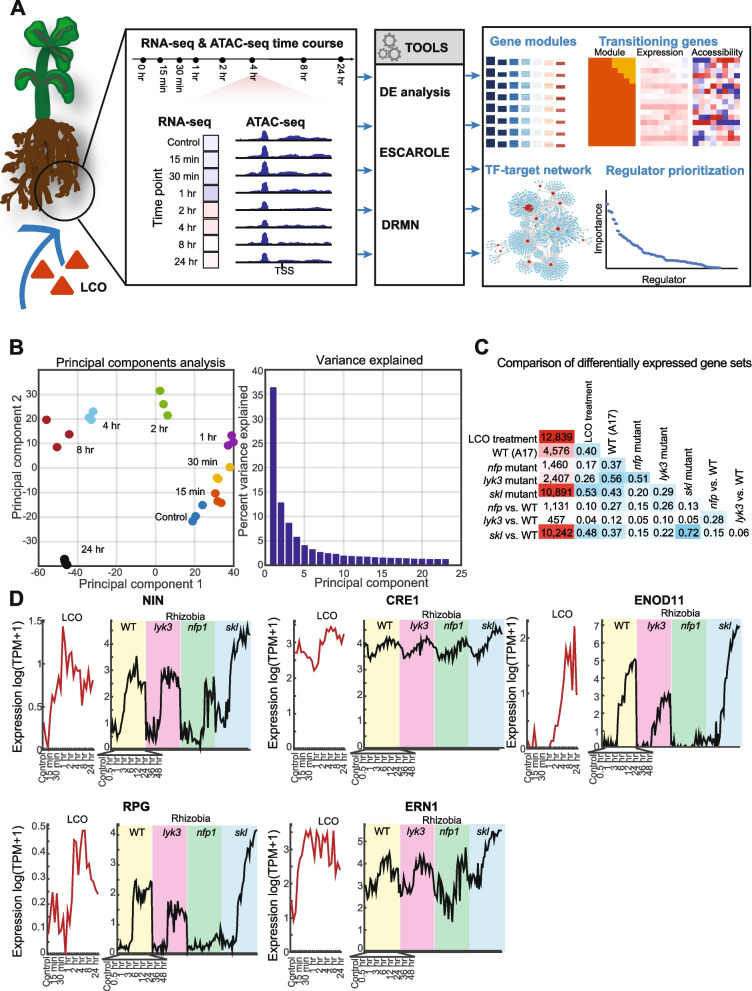
Overview of study. **A** Medicago roots were subjected to LCO treatment, followed by time course profiling of ATAC-seq and RNA-seq measurements. The data were analyzed using computational tools for differential gene expression analysis (DE analysis), time course gene expression analysis (ESCAROLE), and integrative analysis of RNA-seq and ATAC-seq time course (DRMN). Outputs from these tools were used to find gene modules, transitioning genes, TF-target interactions, and prioritize regulators. **B** Principal component analysis (PCA) of expression time course showing grouping and ordering of the (3) biological replicates per time point. Principal components 1, 2, and 3 explain ~50% of the variation. **C** Similarity scores (*F*-score) between the differentially expressed genes (DEG) set obtained in this study (LCO treatment) and DEG sets identified from previously published time-course data under rhizobium treatment from Larrainzar et al. For the latter data, DEGs were called with respect to control for each time course (rows and columns corresponding to WT, *nfp*, *lyk3*, *skl*) and with respect to WT at each time point for each mutant strain (rows and columns with “vs. WT” labels). **D** Expression patterns of known nodulation and symbiosis genes (*NIN*, *CRE1*, *ENOD11*, *RPG*, and *ERN1*) in our dataset (LCO treatment) and in the four rhizobia treatment time courses from Larrainzar et al. (WT, *nfp*, *lyk3*, *skl*). The systematic names for the shown genes are MtrunA17Chr5g0448621 (NIN), MtrunA17Chr8g0392301 (CRE1), MtrunA17Chr3g0082991 (ENOD11), MtrunA17Chr1g0197491 (RPG), and MtrunA17Chr7g0253421 (RPG)

The extent of chromatin accessibility change and impact on transcriptional regulation in rhizobial infection, colonization, and nodule development, remains unknown. Thus, we measured temporal changes in the transcriptome (RNA-seq—ribonucleic acid sequencing) and genome-wide chromatin accessibility (ATAC-seq—assay for transposase-accessible chromatin using sequencing) in response to *Sinorhizobium meliloti* LCOs in *M. truncatula* roots (Fig. 1A). To characterize the role of chromatin accessibility and consequent impact on transcriptional dynamics, we applied a novel algorithm, dynamic regulatory module networks (DRMN) [18], to predict gene expression as a function of chromatin accessibility profiles of *cis*-regulatory features. DRMN results suggest that chromatin accessibility and specific TFs play a critical role in regulating the transcriptional dynamics in response to LCOs.

## Results

### Root transcriptome response to LCOs involves genes activated by rhizobia and early nodule development in *Medicago*

We profiled the global transcriptomic changes of rhizobium LCO signaling with RNA-seq in *M. truncatula* using the Jemalong A17 genotype, treated with LCOs purified from *S. meliloti*. An LCO concentration of 10^−8^ M was used, as in previous studies [19, 20]. Samples were analyzed for control (*t* = 0 h) and seven time-point conditions after treatment (15 and 30 min; 1, 2, 4, 8, and 24 h). Principal component analysis (PCA) showed clustering of biological replicates and time-dependent ordering, the first component explaining ~36% of variation (Fig. 1B, Additional file 1: Figure S1). Comparison of expression levels at each time point (relative to control, *t* = 0 h) revealed 12,839 differentially expressed (DE) genes with significant change in expression (adjusted-*P* < 0.05), including 7540 and 7051 upregulated and downregulated at one time point relative to control, respectively (Additional file 1: Figure S2A). When comparing any pair of time-points we identified 17,391 DE genes in total (Additional file 1: Figure S2A). Both the statistics (Additional file 1: Figure S2B) and heat-maps of DE genes (Additional file 1: Figure S2C, D) present clear patterns of temporal change.

To corroborate these results with previous work on transcriptome dynamics of symbiosis, the identified DEGs were compared to DEGs identified from a published time course data of *M. truncatula* roots inoculated with rhizobium from Larrainzar et al. [12] (see Additional file 1: Figure E-G). Comparisons were made to DEGs in the following genotypes: Jemalong A17 wild type, LCO-insensitive *nfp* mutant, infection *lyk3* mutant, and LCO-hypersensitive *skl* mutant. The highest similarity, measured by *F*-score, to our DEG set was for the mutant genotype most sensitive to LCOs, *skl* (0.53), and the wild-type (WT) strain (0.40). Marker genes for rhizobium-induced nodulation were upregulated (compared to *t* = 0 h), including *NIN* (*nodule inception*, induced after 15 min, with a maximum induction at *t* = 1 h), *CRE1* (*cytokinin response 1*, at 4 h, 8 h, and 24 h), *ENOD11* (*early nodulin 11*, highly induced at 8 and 24 h), *RPG* (*rhizobium-directed polar growth*, at 4 h), and *ERN1* (*ethylene responsive factor required for nodulation 1*, induced as early as *t* = 15 min, Fig. 1D). The similarity was lowest for the *lyk3* (0.26) and *nfp* (0.17) mutants (Fig. 1C, see Additional file 1: Figure S2E, F). Furthermore, when comparing individual time points, DE gene sets are most similar for the later time points (Additional file 1: Figure S2G). While our DEGs had the greatest overlap with the *skl* genotype DEGs, we detected more DEGs compared to Larrainzar et al, likely due to differences in growth conditions (aeroponics versus agar plates) and treatment (purified LCOs versus *Sinorhizobium medicae*), both inducing a strong LCO response.

**Fig. 2:**
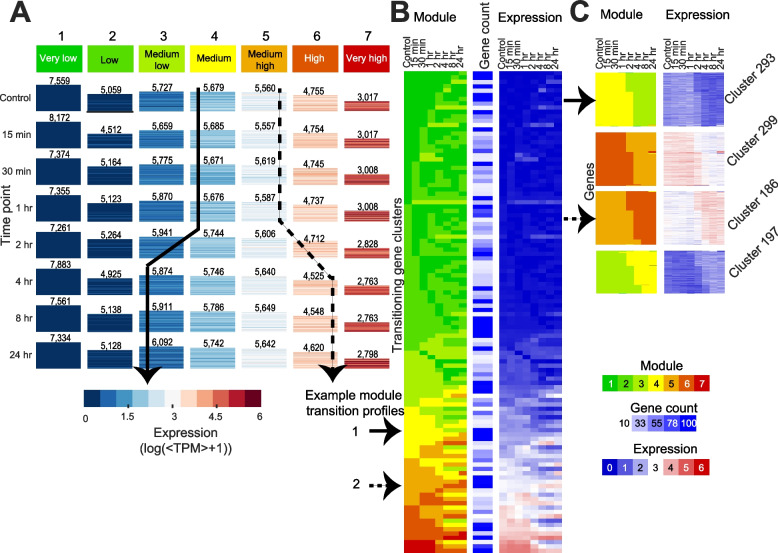
Transcriptome dynamics in response to LCOs. **A** ESCAROLE results for seven modules, based on transcript abundance data. Each heatmap includes genes assigned to that module at that time point, and the height of each heatmap corresponds to the number of genes (inset numbers). **B** The module assignment heatmap depicting typical gene expression trends obtained by hierarchical clustering of gene module profiles into transitioning gene sets. Shown are the mean module assignments, number of genes in each set, and expression levels at each time point for each cluster. Arrows indicate two example trends of expression change. **C** Examples of transitioning gene sets showing gene expression upregulation or downregulation, enriched for genes implicated in nodulation such as defense response to bacterium (cluster 293) and meristem growth (cluster 186)

To examine more complex transcriptome dynamics beyond pairwise DE analysis associated with LCO response, we applied ESCAROLE, a probabilistic clustering algorithm designed for non-stationary time series [21]. The expression data were clustered into seven modules at each time point (very low, low, medium-low, medium, medium-high, high and very high expression, Fig. 2A). Seven modules maximized the log-likelihood and silhouette index (Additional file 1: Figure S3A, B). Next, 12,261 transitioning genes (those changing module assignment over time) were identified, including several implicated in symbiosis (Additional file 1: Figure S3C). Transitioning genes with similar dynamics were clustered using hierarchical clustering, identifying 112 clusters (> = 10 genes each) (Fig. 2B) including 11,612 genes (Methods). Among clusters representing downregulation of expression over time, several were enriched for Gene Ontology (GO) processes implicated in defense responses to bacterium (cluster 293, downregulated from 2–4 h), and the biosynthesis of plant hormones involved in the suppression of nodulation (Fig. 2C). For instance, cluster 299 (downregulated after 2 h) is enriched (hypergeometric test *q* < = 0.05) for jasmonic acid (JA) biosynthesis and JA response genes, including *Coronatine insensitive 1* (*COI1*), which forms part of the JA co-receptor complex for the perception of the JA-signal [22]. Among the gene clusters upregulated over time, several are implicated in early stages of symbiosis and nodule development. For instance, cluster 186 (induced 2–4 h after LCO treatment; Fig. 2C) is enriched in genes implicated in the regulation of meristem growth, including an *Arabidopsis trithorax 3* (*ATX3*) homolog (MtrunA17Chr4g0005621) and a *lateral organ boundaries domain* (*LBD*) transcription factor (MtrunA17Chr4g0043421). *ATX3* encodes an H3K4 methyltransferase [23], and LBD proteins are characterized by a conserved lateral organ boundaries (LOB) domain and are critical regulators of plant organ development [24], including lateral roots and nodules [25]. This cluster also contains *EPP1* and the cytokinin receptor *CRE1*, both positive regulators of early nodule symbiosis and development [26, 27]. Other essential regulators of LCO signaling are also found in clusters exhibiting induction under LCO treatment (Additional file 1: Figure S3D), such as *DMI1* (cluster 197, Fig. 2C), *NIN* (cluster 205), *NF-YA1* (cluster 177), and the marker of LCO perception *ENOD11* (cluster 296). Together, the DE and ESCAROLE analysis showed that *M. truncatula* response to LCOs is characterized by complex expression dynamics recapitulating several known molecular features of this process.

### LCO treatment causes genome-wide changes in chromatin accessibility

To study chromatin accessibility changes in a genome-wide manner in response to LCOs, we performed ATAC-seq on samples at all time points matching our RNA-seq time course. Overall, 54–235 million paired-end reads were obtained for each sample, with 46–75% mappable to the (v5) reference genome (Additional file 1: Figure S4). Moreover fragment distributions in ± 1 kbp of the transcription start site (TSS) were examined (Additional file 1: Figure S5, see Methods) and favorably compared to previously published *M. truncatula* ATAC-seq data [28] (Additional file 1: Figure S6).

**Fig. 3:**
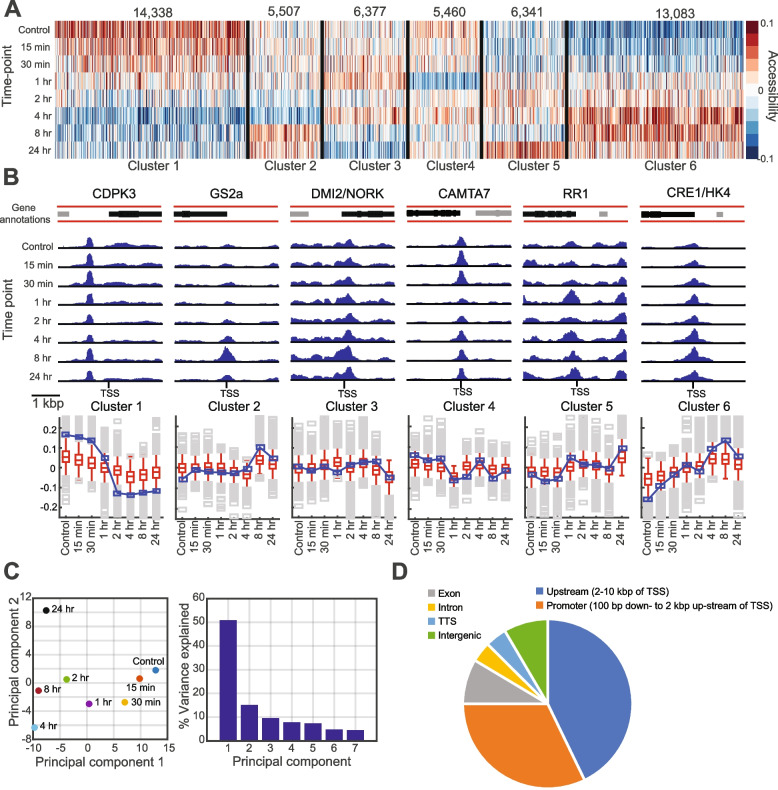
Chromatin accessibility data exploratory analysis. **A** Clustering of promoter accessibility profiles in the LCO treatment time course. **B** IGV track and profiles of coverage for the promoter regions (± 2 kbp of TSS) of genes involved in root nodulation, representative of each cluster (upper panel). Gene annotation track (top) denotes the gene of interest (black) and neighboring genes (gray). **C** PCA results for the same promoter accessibility data. **D** Distribution of genomic regions for the universal ATAC-seq peaks

We next evaluated aggregated chromatin accessibility in gene promoter regions, defined as ± 2 kbp around the TSS, across time. To quantify promoter accessibility, we obtained the mean per base pair (per-bp) read coverage within each region, for each time point. For each time-point, the log-ratio of per-bp read coverage in each promoter was taken relative to the global mean of per-bp coverage, quantile normalized across time points. High consistency was found between promoter signals between technical replicates from each time point based on Pearson’s correlation (Additional file 1: Figure S7, (Pearson correlation 0.965–0.990)) and PCA (Additional file 1: Figure S8A). We partitioned the resulting 51,007 gene promoter accessibility profiles into six characteristic patterns (clusters) using k-means clustering (Fig. 3A, Additional file 1: Figure S8B). Clusters 1 (14,338 genes) and 6 (13,083 genes) exhibit general patterns of decrease and increase in accessibility, respectively, whereas clusters 2–5 (5460–6377 genes) present more transient variation. The correlation of accessibility between time points suggests an overall reorganization of promoter accessibility 1–2 h after the treatment (Additional file 1: Figure S8C). The temporal change in accessibility is evident for the promoters of several nodulation genes, including *CRE1*, *CYCLOPS*, and *EIN2* (Fig. 3B, Additional file 1: Figure S8D, E; prepared with the Integrative Genome Viewer—IGV) [29]. PCA of the promoter signals showed time-dependent variation (Fig. 3C, Additional file 1: Figure S8A), with the first component explaining > 50% of the variance.

We called peaks for each time point using the Model-based Analysis of ChIP-Seq version 2 (MACS2) algorithm [30] (Additional file 1: Figure S9A) and merged peaks across time points with at least 90% overlap into *universal* peaks (Additional file 1: Figure S9B). Chromatin accessibility peaks showed a similar genomic distribution across time points, with 32.1% of peaks located within 2 kbp upstream and 100 bp downstream of a gene TSS (Additional file 1: Figure S9A-C) and spanning 50.4 Mbp (11.7%) of the *M. truncatula* (v5) genome. As with the promoter accessibility, clustering accessibility profiles of universal peaks identified distinct patterns of temporal change (Additional file 1: Figure S9D, E). Several of the clusters were associated with known TF motifs (Additional file 1: Figure S9F) and specific types of genomic regions. For example, clusters 1, 2, and 7 had higher proportions of intergenic peaks (hypergeometric test *P* < 0.05, Additional file 1: Figure S9G). Genes mapped to peaks associated with cluster 2 were enriched for photosynthesis and protein-chromophore linkage (hypergeometric test *q* < 0.05). Collectively, these results suggest that LCO treatment had a genome-wide impact on chromatin accessibility, prospectively associated with simultaneous change in gene expression.

### Chromatin accessibility is correlated with transcriptional dynamics of nodulation genes

**Fig. 4:**
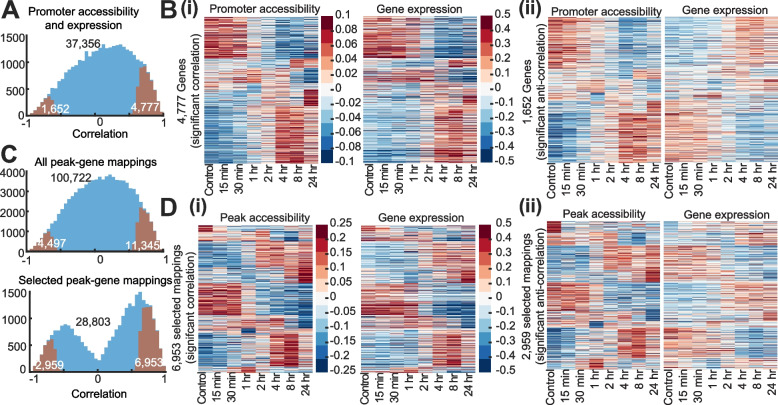
Correlation between chromatin accessibility and gene expression. **A** Histogram of Pearson’s correlation of all (blue) and significantly correlated (orange) promoter accessibility and gene expression pairs. The number of pairs are indicated with inset numbers. **B** Clusters of promoter accessibility and gene expression for significant (*P*<0.05) (i) positive and (ii) negative correlation relative to random. **C** Histograms of correlation for all (blue) and significantly correlated peak and gene pairs (orange) and associated statistics. The upper histogram includes all mapped peak-gene pairs, while the lower includes only the maximally correlated peak for each gene (below). **D** Clustered peak accessibility and corresponding expression profiles for significantly positively (i) or negatively (ii) correlated gene-peak mappings

We evaluated the relationship between gene expression and promoter chromatin accessibility ± 2 kbp around the TSS and universal peaks within 10 kbp upstream and 1 kbp downstream of a gene TSS. Correlating promoter accessibility and gene expression profiles identified 6429 genes with significant correlation (Fig. 4A, *P* < 0.05 relative to random permutation): 4777 with positive correlation and 1652 with negative correlation (Fig. 4B), representing 17.2% of the 37,356 genes analyzed. Among these were 36 genes with known roles in symbiosis (Additional file 1: Figure S8D), including *ERN1*, *CRE1*, *LYK10/EPR3*, *SKL/EIN2*, and *IDP3*/*CYCLOPS* with positive correlation, and *LYK8*, *ERN2*, *CAMTA3*, and *CAMTA4* with negative correlation. We next examined significantly correlated genes (Fig. 4A) and visualized those expression and accessibility profiles as ordered by the promoter accessibility clusters (Fig. 3A), separately for positive and negative correlation (Fig 4B). This revealed robust patterns of consistency between promoter accessibility and expression.

Correlating accessibility of universal peaks centered within 10 kbp upstream to 1 kbp downstream of gene TSSs identified 100,722 peak-gene mappings (out of a total 125,140) associated with 28,803 (of 37,536) expressed genes (Fig. 4C, Additional file 1: Figure S9C and G). Peak accessibility was significantly correlated with gene expression in 15.7% of these pairings (Fig. 4C), comparable to the 17.2% (6429) genes with significant correlation between expression and gene TSS accessibility. When considering each gene and only the most correlated peak (28,803 selected pairs), 34.4% (9912 genes) were significantly correlated, including 56 nodulation genes (Fig. 4D). Of these 9912 genes presenting significant correlation, 5735 (57.9%) do not present significant correlation with the corresponding promoter accessibility, indicating a prominent role for distal regulation (> 2 kbp of gene TSS) for these genes. Such peaks were in general more distal from TSS sites than those that presented significant correlation with corresponding TSS accessibility (Kolmogorov-Smirnov/KS test *P* < 0.05).

Finally, the ESCAROLE-defined transitioning gene clusters exhibited coordinated trends between promoter accessibility and gene expression (Fig. 2B, Additional file 1: Figure S3D). Two thousand five hundred one of the 11,612 (21.5%) transitioning genes that could be clustered exhibited significant correlation between their profiles of expression and promoter chromatin accessibility. These results suggest that chromatin accessibility is an important regulatory mechanism in transcriptional response to LCOs.

### DRMN integration of ATAC-seq and RNA-seq data identifies key regulators that determine gene expression dynamics in response to LCOs

To better understand how chromatin accessibility contributes to transcriptional changes in rhizobia-plant symbiosis, we applied dynamic regulatory module networks (DRMN) [18] to integrate the RNA-seq and ATAC-seq time course data. DRMN extends the ESCAROLE analysis (which examined only the transcriptome) by modeling the relationship between variation in accessibility and gene expression. DRMN predicts gene expression as a function of regulatory features [31] by first grouping genes into modules based on expression levels (similar to ESCAROLE) and then learning a regulatory program for each module. DRMN uses regularized regression and multi-task learning to incorporate the temporal nature of a data set [32] to simultaneously learn regression models for each module in each time point.

**Fig. 5:**
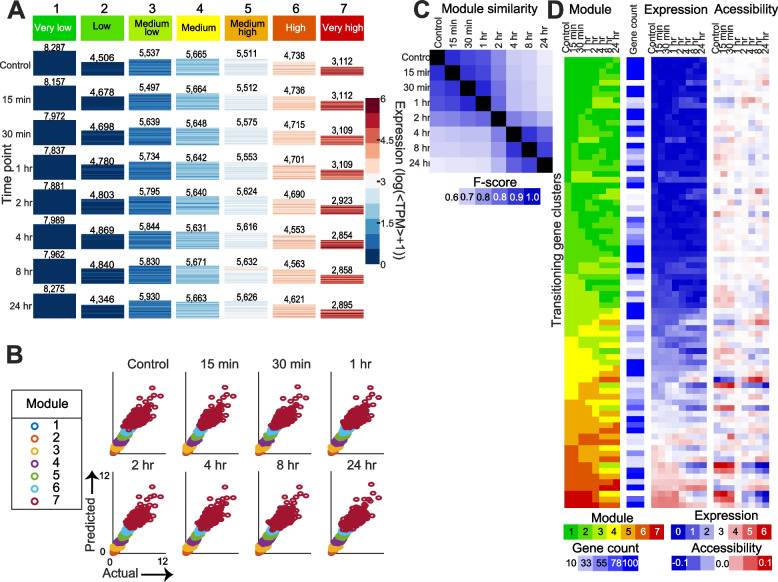
Dynamic regulatory module network (DRMN) analysis. **A** Heatmap of DRMN inferred expression modules across the time course. Each heatmap corresponds to an expression module for each time point, the size of the heatmap indicating the number of genes assigned to that module (listed on top). **B** Scatter plots of actual and predicted expression values. **C ***F*-score similarity of DRMN modules across time points. **D** DRMN transitioning gene sets. Shown are the mean DRMN module assignment, number of genes, mean expression levels, and mean promoter accessibility levels for each transitioning gene set (rows) across time (columns)

We applied DRMN with seven expression modules using two types of features (Fig. 5A, Additional file 2: Tables S1-S4): (1) the aggregated signal of ATAC-seq reads in gene promoters (± 2 kbp of the TSS) and (2) the ATAC-seq signal in genomic coordinates of known motifs within − 10 kbp and + 1 kbp of the TSS. Both feature types represent chromatin accessibility, but the first is independent of the presence of known motifs, whereas the second captures the accessibility of motif sites. Motif features were based on the CisBP v1.2 database for *M. truncatula* [33] and curated motifs of several known regulators of root nodulation, including CYCLOPS, NSP1, NIN, and the nitrate response *cis*-element (NRE). Hyper-parameters for DRMN were selected using a grid search and quality of inferred modules (Additional file 1: Figure S10A). The DRMN modules represent statistically different expression levels (Additional file 1: Figure S10B, Kolmogorov-Smirnov test *P* < 10^−300^). To assess the extent to which DRMN captures variation in expression, we correlated predicted and measured expression levels (Fig. 5B, Additional file 1: Figure S10A, C). The mean Pearson correlation of predicted and measured values per module was 0.26–0.46 (Additional file 1: Figure S10C) across all modules and time points, the least expressed module being most difficult to predict. Comparing the genes in each module showed that the modules are more similar (*F*-score 0.88–0.94, Fig. 5C) before and after 2 h, than across this time point (*F*-score < 0.80), suggesting a significant module reorganization at ~2 h. This is consistent with the general reorganization of promoter accessibility ~1–2 h after the treatment and global expression correlation around 2 h (Additional file 1: Figure S8C). We additionally tested the modules for enrichment of known motifs (Additional file 1: Figure S11, Additional file 2: Table S3) and Gene Ontology (GO) processes (Additional file 1: Figure S12). Several regulators (e.g., KNOX and EDN transcription factor family members) and processes relevant to symbiosis were identified, including nodule morphogenesis, root-hair elongation, and the MAPK cascade, as well as others relating to gene regulation and chromatin organization. Finally, we used the DRMN module assignments to define transitioning gene sets (Fig. 5D, Additional file 1: Figure S13A), similar to those from ESCAROLE (Fig. 2B, Additional file 1: Figure S13B). We identified 79 transitioning gene clusters including 10,176 genes, of which 5332 (>50%) were differentially expressed with DESeq (hypergeometric-test overlap adjusted-*P* < 0.05), and (8398) 77% were identified in ESCAROLE, indicating consistency between the analyses.

**Fig. 6:**
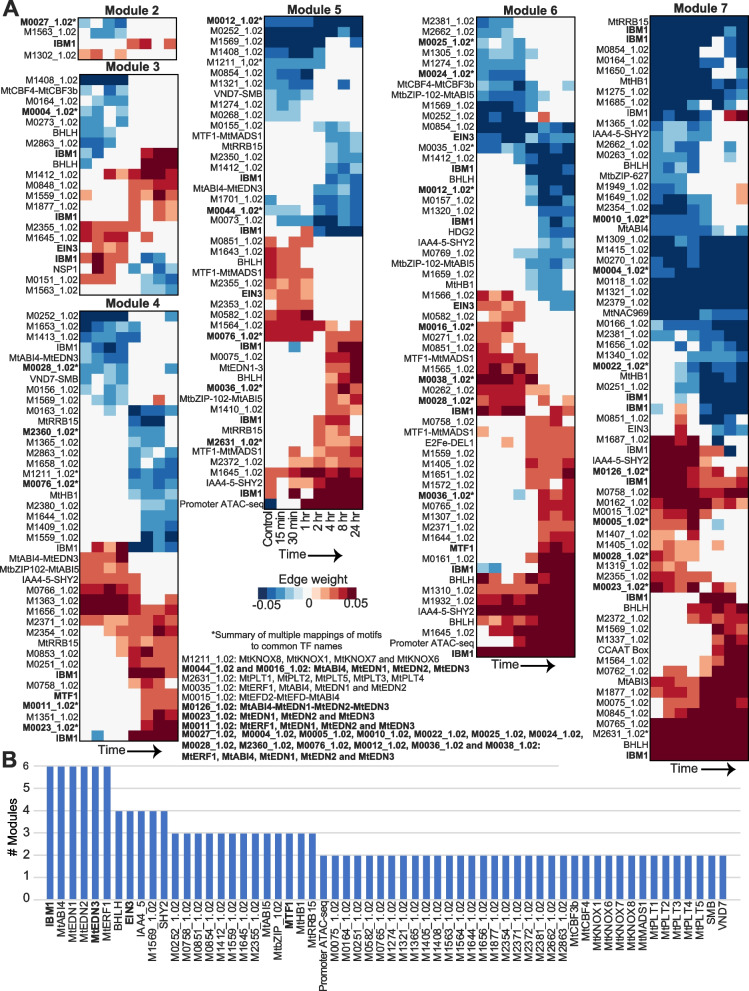
Regulator prioritization results. **A** DRMN regulator regression weights that meet a *T-*test criterion of significant change (*P* < 0.05) between 0–1 h and 2–24 h. CisBP motif IDs mapped to > = 3 common names (*) are summarized separately (bottom center). **B** Regulators prioritized based on the frequency with which they are selected across modules with the *T*-test criteria. Labels of motifs mapped to IBM1, EDN3, MTF1, and EIN3 discussed in the text are in bold in both panels

We used the DRMN results to prioritize regulators that shape transcriptional response to LCOs. Specifically, we identified regulators whose regression coefficient changed significantly (*T*-test *P* < 0.05) between 0–2 and 4–24 h, corresponding to the reorganization of expression modules (Fig. 5C). According to this criterion chromatin accessibility of gene promoters was an important predictor of expression for highly expressed genes (“Promoter ATAC-seq” for modules 5 and 6, Fig. 6A). We also identified the TFs IBM1 (increase in BONSAI methylation 1), ERF1 (ethylene response factor 1), EDN1-3 (ERF differentially regulated during nodulation 1, 2, and 3), EIN3 (ethylene insensitive 3), SHY2 (short hypocotyl 2), ABI4-5 (abscisic acid-insensitive 4 and 5), MTF1 (MAD-box transcription factor 1), and MtRRB15 (type-B response regulator 15), as well as several markers of meristem cells, KNOX and PLT (PLETHORA) protein families as important regulators (Fig. 6B, Additional file 1: Figure S11).

### Identification of the targets of DRMN-prioritized regulators

**Fig. 7:**
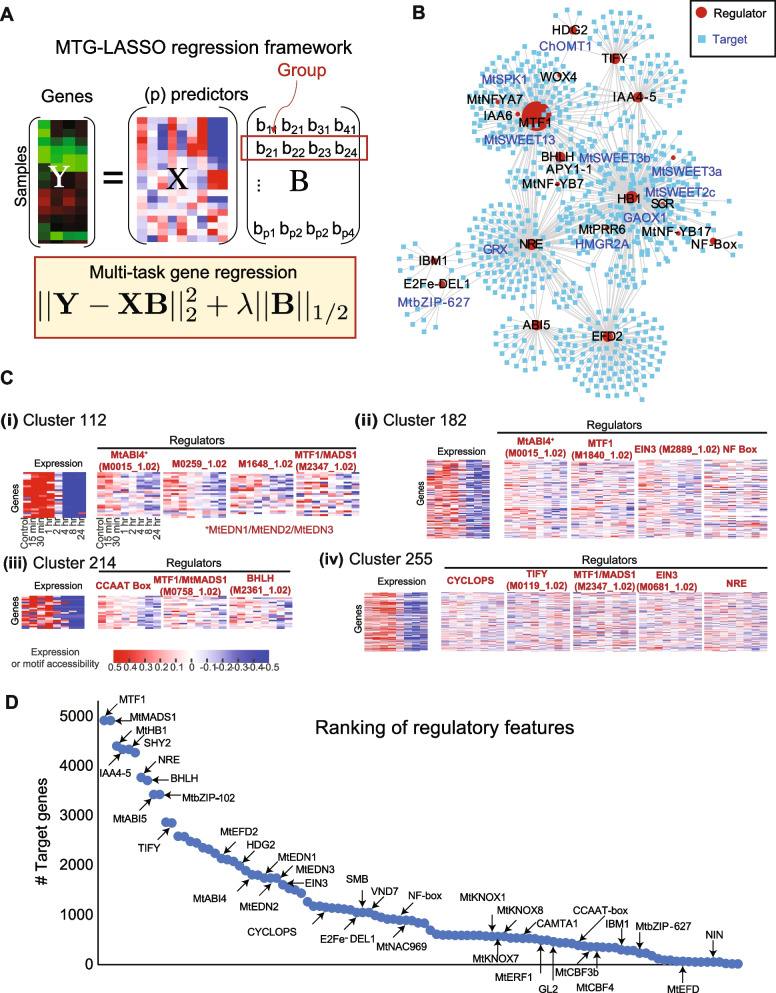
Multi-task group LASSO (MTG-LASSO) to predict regulators of transitioning genes. **A** MTG-LASSO was applied to infer significant regulatory features for each transitioning gene set. Shown is a model of predicting expression (Y) for a set of genes using the predictor features (X) of the genes and coefficients (B). Each gene (column of Y) is a task and each row of B corresponds to the regression of a predictor for all genes. MTG-LASSO picks the same regulators for all genes in a set but allows for different regression weights. The regression weights for a regulator (row) is a group. **B** Visualization of the top 1000 predicted TF-gene network edges, ranked by regression weight magnitude from MTG-LASSO. **C** Example transitioning gene sets showing corresponding gene expression and motif accessibility profiles for regulators of interest (IBM1, MTF1, EIN3, EDN3). For each cluster, we show genes with significant change in accessibility between 0–2 and 4–24 h (*T*-test *P*-value < 0.05) for at least one regulatory feature per cluster. **D** Ranking of all regulators selected in the MTG-LASSO-based regulatory network. Regulators are ranked by the number of predicted targets. The motifs that were mapped to a common name are shown. The ranking highlights regulators identified at the DRMN module level (Fig. 6) and additional regulators like TIFY and CYCLOPS

DRMN identified regulators of gene expression dynamics in response to LCOs. Next, we aimed to identify their gene targets. Expression-based network inference is commonly used to define regulator-gene relationships [34] but is challenging with only 8 time-points. To address this, we used the DRMN transitioning gene sets and regulatory motifs selected by a regularized regression method, multi-task group LASSO (MTG-LASSO, where LASSO stands for least absolute shrinkage and selection operator) to define the targets of a gene (Methods). This approach modeled the variation in expression of each of the 79 transitioning gene clusters using a structured sparsity approach, multi-task group LASSO (MTG-LASSO) (SLEP v4.1 package [35], Fig. 7A, Additional file 2: Table S4) to identify regulators (motifs/TFs) for each of the transitioning gene clusters. Here, the same feature data from the DRMN analysis was used. We determined MTG-LASSO parameter settings for all 79 transitioning gene sets, identifying 33 with significant regulatory motif associations (Additional file 1: Figure S14). This generated 122,245 regulatory edges connecting 126 regulatory motifs to 5978 genes (Fig. 7B). Several gene sets exhibit consistent downregulation of expression and corresponding reduction in accessibility of predicted regulatory motifs between 0–2 and 4–24 h (Fig. 7C). For example, gene set 214 (57 genes) shows downregulation of gene expression and reduced motif accessibility (after 4 h) for multiple TFs: MTF1 and BHLH (Fig. 7C). Similarly, gene set 182 was predicted to be regulated by EDN3, MTF1, EIN3, and NF-Box motif and exhibited correlated trends between gene expression and regulatory feature accessibility (Fig. 7C). We prioritized regulators based on the number of targets they were predicted to regulate and found several known and novel regulators in the top-ranking set), such as ERF1 (ethylene response factor 1), EDN1-3 (ERF differentially regulated during nodulation 1, 2, and 3), EIN3 (ethylene insensitive 3), SHY2 (short hypocotyl 2), and MTF1 (MAD-box transcription factor 1) (Fig. 7D).

### *EIN3* and *ERF1* are important regulators of root nodule symbiosis in *M. truncatula*

**Fig. 8:**
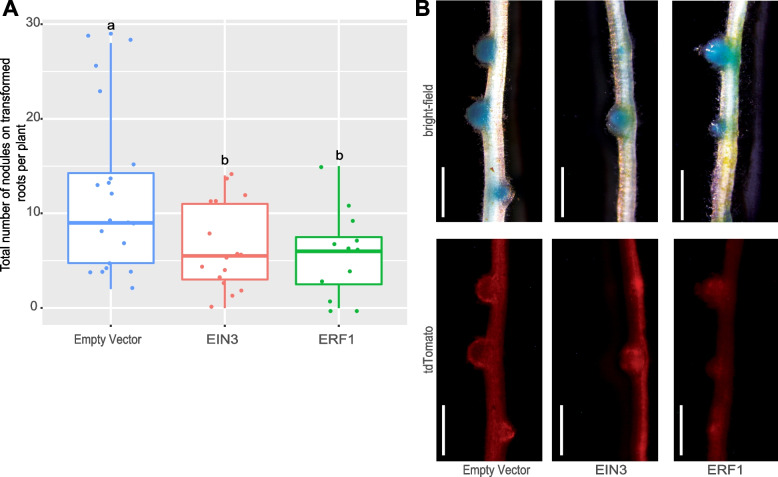
RNAi knockdown of *EIN3* and *ERF1* reduced the number of nodules on *M. truncatula* plants. **A** Data for empty vector control, and EIN3 and ERF1 knock down roots (*n* = 20, 16, and 13 replicates respectively) were analyzed by ANOVA followed by Tukey’s HSD test for multiple comparisons. Box plots not connected by the same letter are significantly different (*P* < 0.05). One extreme outlier (29 nodules) was excluded in the *MtrunA17Chrg0186741* (*ERF1*) experiment. **B** Images of nodules on subtending root supporting the effectiveness of RNAi. Blue color (top) indicates the rhizobial infection (*S. meliloti* constitutively expressing lacZ), and the red fluorescence marker (bottom) identifies transgenic roots (white scale bar = 1 mm)

To experimentally test the involvement of DRMN prioritized transcription factors in root nodule symbiosis, we selected three TFs, *EIN3*, *ERF1*, and *IAA4-5* which were among the DRMN selected regulators (Fig. 7D). We knocked down the expression of the corresponding genes by RNAi and examined the nodulation phenotype in composite *M. truncatula* plants (Methods). Knockdown of *MtrunA17Chr5g0440591* (*EIN3*) and *MtrunA17Chr1g0186741* (*ERF1*) significantly lowered the number of nodules produced on the RNAi roots (Fig. 8A, Additional file 1: Figure S15A, *P*<0.05 from an ANOVA test followed by Tukey’s HSD test post hoc). Knockdown of *MtrunA17Chr1g0166011* (*IAA4-5*) did not alter nodulation relative to the empty vector (EV) control (Additional file 1: Figure S15B, Additional file 2: Table S5). These nodules were all colonized by *S. meliloti* (Fig. 8B). Together, these results validate the role of *MtrunA17Chr5g0440591* (*EIN3*) and *MtrunA17Chr1g0186741* (*ERF1*) in rhizobium-legume symbiosis, as predicted by DRMN.

## Discussion

The enormous economic and environmental cost of plant nitrogen fertilization motivates efforts towards identifying molecular mechanisms underlying legume perception of nitrogen-fixing bacteria and nodule development. We dissected the gene regulatory network in *M. truncatula* roots in response to *S. meliloti* LCOs by jointly profiling the temporal changes in the transcriptome and chromatin accessibility and integrating these data computationally. Extensive changes in the transcriptome are known to occur in *Medicago* roots in response to rhizobia signals, and we show these changes are accompanied and facilitated by extensive chromatin remodeling. While the overall percentage of accessible chromatin regions remained similar across our time course experiment, regions of accessibility underwent a dramatic shift 1–2 h after treatment. This remodeling appears to anticipate the development of root nodules, which requires stringent temporal and spatial control of gene expression. Chromatin accessibility of gene promoters notably also emerged as a significant predictor of gene expression (Fig. 6). These changes in chromatin accessibility enable and enhance the transcriptional changes required for nodule development by providing regulators access to promoters that may be inactive in other stages of plant development. Correlation was additionally observed between gene expression and promoter chromatin accessibility profiles of several essential regulators of nodulation, including *ERN1*, *CRE1*, *SKL/EIN2*, *IDP3/CYCLOPS*, and *ERN2*. Close coordination between chromatin accessibility and gene expression in LCO response is likely essential for root nodule development.

We applied novel methods for time-series analysis, ESCAROLE and DRMN [36], to model temporal changes in gene expression and chromatin accessibility. ESCAROLE enabled us to characterize the transcriptional dynamics beyond pairwise differential expression analysis, while DRMN allowed us to jointly analyze transcriptome and chromatin dynamics and predict which transcription factors (TFs) are most important for expression dynamics. Consistent with the theme of chromatin reorganization under LCO treatment response, DRMN identified IBM1 as a critical regulator. IBM1 encodes a JmjC domain-containing histone demethylase that catalyzes the removal of H3K9 methylation and di-methylation in Arabidopsis [37]. DRMN also identified regulatory genes involved in hormone responses in the early steps of symbiosis and nodule formation such as ethylene (*ERF1*, *EDN1-3*, and *EIN3*) and ABA (*ABI4-5*). EIN3 is a transcription factor mediating ethylene-regulated gene expression and morphological responses in *Arabidopsis*. The role of EIN3 in rhizobium-legume symbiosis or LCOs signaling remains uncharacterized, but *sickle* (*skl*) mutants for an *EIN2* ortholog develop more infection threads and nodules and respond more to LCOs than wild-type plants, and ethylene treatment inhibits LCO signaling and nodule formation [38]. ABI4 and ABI5, basic leucine zipper transcription factors implicated in several plant functions, coordinate LCO and cytokinin signaling during nodulation in *M. truncatula* [39]. DRMN also identified regulators associated with the hormones involved in the nodule initiation, auxin (*SHY2*), and cytokinin (*MtRRB15*). *SHY2*, a member of the Aux/IAA family, plays a critical role in cell differentiation at root apical meristem and is activated by cytokinin [40, 41]. *SHY2* was proposed as a candidate for nodule meristem regulation and differentiation after showing a very localized expression pattern in the nodule meristematic region [42]. Also related to nodule meristem initiation, KNOX TF-family members and PLT1-5 were predicted as regulators of gene expression in response to LCOs. *MtPLT* genes (*MtPLT1-5*) are part of the root developmental program recruited from root formation and control meristem formation and maintenance for root and nodule organogenesis [43]. We experimentally validated two of our regulators *EIN3* and *ERF1* using RNAi in *M. truncatula* and showed a significant effect in nodule formation. Prior work of Asamizu et al. [44] independently supports the observation of the *ERF1* ortholog as an effector of nodule development in *L. japonicus*, where the number of nodules was likewise reduced in a similar RNAi experiment. Their findings suggest *ERF1* is induced by rhizobium on a 3 to 24 h time scale, echoing the observed time scale of chromatin reorganization in *M. truncatula* in our work. Recent work of Reid et al. [45] emphasizes an early, positive role of ethylene in rhizobium-legume symbiosis in *L. japonicus*, which supports why we observe ethylene-related TFs having a positive impact on nodulation, unlike the ethylene insensitive *skl* mutation [38]. The exact mechanisms by which these genes regulate rhizobium-legume symbiosis can be explored in future research.

Our analysis predicted genome-wide targets for transcription factors, including novel regulators identified by DRMN and previously known regulators of root nodulation, such as *NIN*, *NF-YA1/NF-YB1*, and *CYCLOPS*. For example, MTG-LASSO analysis predicted *NIN* as a direct target of SHY2 and MTF1, and *FLOT4*, required for infection thread formation, as a target of IBM1 [46]. Among known regulators, MTG-LASSO indicated that *ARF16a* and *SPK1* are targets of NF-Y TFs. ARF16a and SPK1 control infection initiation and nodule formation [1]. Several NF-Y genes (NF-YA5 and NF-YB17) were identified as regulated by CYCLOPS. These predicted regulatory relationships can be tested with future validation experiments and uncover key mechanisms underlying the regulation of gene expression in LCO response

## Conclusions

The regulatory mechanisms underlying plant-microbe symbiotic relationships remain poorly characterized. Here, we present a novel dataset that profiles the concurrent changes in transcriptome and chromatin accessibility in the model legume, *Medicago truncatula*, in response to rhizobia signal that trigger nodule formation. We have jointly modeled the chromatin and transcriptome time series data to predict the most critical regulators of the response to these signals and that underlie molecular pathways driving nodule formation. Our transcriptomic and accessibility datasets and computational framework to integrate these datasets provide a valuable resource for identifying key regulators for the establishment of root nodulation symbiosis in *M. truncatula* that could inform engineering of nodulation in species unable to establish that symbiosis naturally.

## Methods

### Plant material and treatment

Seeds of wild-type *Medicago truncatula* Jemalong A17 strain (available through the USDA Germplasm Resources Information Network (GRIN)) were sterilized and germinated in 1% agar plates, including 1μM GA3. Plates were stored at 4 °C for 3 days in the dark and placed at room temperature overnight for germination. Seedlings were grown vertically for 5 days on a modified Fahraeus medium with no nitrogen [47], in a growth chamber (24 °C, 16 h light/8h dark cycle, 70 μmol m^−2^ s^−1^ photosynthetic photon flux). LCOs were purified from *S. meliloti* strain 2011 as described previously [48]. Next, seedling roots were immersed in a solution of purified LCOs (10^−8^ M) or 0.005% ethanol solution (control) for 1 h. Roots were cut and immediately used for nuclei extraction and generation of ATAC-seq libraries (see below) or snap-frozen in liquid nitrogen for posterior RNA isolation and sequencing. Roots were collected at 0 h (control), 15, 30 min, 1, 2, 4, 8, and 24 h after LCO treatment. Roots from seven plants were pooled for each of three biological replicates used in RNA sequencing, while roots from 15 plants were pooled for one replicate used in ATAC-seq, in each time point of the experiment.

### ATAC-seq library preparation and sequencing

For ATAC-seq library preparation, we followed the protocol described previously [49] with modifications. Before nuclei isolation, all materials were precooled to 4 °C. Briefly, roots were chopped for 2 min in 1 ml of pre-chilled lysis buffer (15 mM Tris-HCl pH7.5, 2mM EDTA, 20 mM NaCl, 80 mM KCl, 0.5 mM spermine, 15 mM 2-ME, 0.15 % TritonX-100) in a cold room. This step was repeated four times with a 1 min interval between repetitions. The homogenate was filtered through one layer of pre-wetted Miracloth, loaded on the surface of a 2 mL dense sucrose buffer (1.7 M sucrose, 10 mM Tris-HCl pH8.0, 2 mM MgCl_2_, 5 mM 2-ME, 1 mM EDTA, 0.15 % Triton X100), and centrifuged (2400 g, 20 min at 4 °C). The supernatant was removed, and the nuclei were resuspended in 500 μl of lysis buffer and then filtered in 70 μm and 40 μm filters consecutively. The nuclei were then collected by centrifuging the solution at 1000*g* for 5 min at 4 °C. After washing with 950 μl 1×TAPS buffer (10 mM TAPS-NaOH, pH8.0, 5 mM MgCl_2_), the samples were centrifuged again at 1000*g* for 5 min at 4 °C. The supernatant was removed, leaving the nuclei suspended in approximately 10 μl of solution. Next, 1.5 μl of Tn5 transposase (Illumina FC-121-1030), 15 μl of Tagmentation buffer, and 13.5 μl of ddH_2_0 were added to the solution. The reaction was incubated at 37 °C for 30 min. The product was purified using a QIAGEN MinElute PCR Purification kit and then amplified using Phusion DNA polymerase. One microliter of the product was used in 10 μl qPCR cocktail with Sybr Green. Cycle number X was determined as the cycle were the ¼ of the maximum signal was reached. Then, we amplified the rest of the product in a Phusion (NEB) PCR system with X-2 cycles (10 to 15 cycles, 50 μl of reaction). Amplified libraries were purified with AMPure beads (Beckman Coulter), and library concentrations were determined using a Qubit. Sequencing was carried out in an Illumina HiSeqX (2 × 150 cycles) at the HudsonAlpha Institute for Biotechnology (Huntsville, AL, USA).

### RNA-seq library preparation and sequencing

For each RNA extraction, roots from 7 plants were pooled and ground while keeping the sample frozen. RNA extraction was performed as described previously [50]. Libraries were prepared using 1 μg of RNA in the NEBNext® Ultra™ Directional RNA Library Prep Kit following the supplier’s instructions (New England Biolabs, Ipswich, MA, USA). Sequencing was carried out with an Illumina HiSeq3000 (2 × 100 cycles) at the Interdisciplinary Center for Biotechnology Research at the University of Florida (Gainesville, FL, USA).

### RNA-seq data pre-processing and quality control

Between 8.7 and 17.9 million, 2 × 100 bp reads were obtained after sequencing the 24 RNA-seq libraries. Reads were aligned with Kallisto [51] to the *M. truncatula* transcriptome (v5, [52], Additional file 1: Figure S1). The average of the alignment rates across time points was 87–95%. A total of 37,536 genes were detected with non-zero expression at any of the time points. The data were processed with SLEUTH [53] for further analysis. Finally, TPM expression values were quantile-normalized and log-transformed before being used as input for further analysis. Principle component analysis was applied to these data in MATLAB (Fig. 1A). For comparative purposes, transcriptome time course data related to root nodulation [12] obtained from the *M. truncatula* wild-type reference accession Jemalong A17 and three mutants (*lyk3*, *nfp*, and *skl/ein2*), were analyzed using the same Kallisto/SLEUTH approach. The 144 samples characterized in that experiment presented alignment rates of 91–96%, except four outliers with rates of 73–88%. Analysis of this data set detected 40,988 genes with non-zero expression, of which 36,298 were in common with the 37,536 identified in the present LCO-treatment experiment (Additional file 1: Figure S1).

### Differential expression analysis of RNA-seq time course and comparison with existing data

DESeq [54] was applied to both the data generated in the present work and previously published data sets for four rhizobial treatment [12]. The expected count matrices of each data set were used as input to the DESeq algorithm, used in a default manner per the author recommendations. For each of the five time-course experiments, we assessed differential expression relative to control (time 0 h) for each later time point (Additional file 1: Figure S2A and S2E, left) as well as between pairs of time points (Additional file 1: Figure S2E). An adjusted *P* threshold of 0.05 was applied to select differentially expressed (DE) genes for each time point in each experiment. Statistics (Additional file 1: Figure S2B) and heat-maps for genes DE relative to control and between (all) time points (Additional file 1: Figure S2C and D) present clear patterns of temporal change.

For the Larrainzar et al. data set [12], we also identified differentially expressed genes between the three mutants (*lyk3*, *nfp*, and *skl/ein2*) relative to the wild-type reference (Jemalong A17) for matched time points (Additional file 1: Fig S2E, right). As in the first analysis the union of genes identified at any time point defined the set of differentially expressed genes for the dataset.

The union of differentially expressed genes across time points was used for comparisons between datasets. We quantified the degree of overlap between DE gene sets with an *F*-score, or harmonic mean, of the fraction of overlapping genes in each set using the union across all time points (Fig. 1C, Additional file 1: Figure S2F) as well as individual pairs of time points (Additional file 1: Figure S2G). For two sets of *N*_*1*_ and *N*_*2*_ genes, respectively, and *N*_*O*_ in common between the two, the *F*-score is defined as:

$$F=2\frac{\frac{N_O}{N_1}\frac{N_O}{N_2}}{\frac{N_O}{N_1}+\frac{N_O}{N_2}}$$

### Expression clustering analysis with ESCAROLE

We analyzed the LCO-treatment time course data with ESCAROLE [21] to characterize the temporal changes in the transcriptome. We included 37,536 genes with at least one non-zero count in at least one of the 24 experiments (3 replicates × 8 time points). Transcriptome data from each time point were grouped by k-means clustering and used as an input module assignment for the ESCAROLE algorithm (Fig. 3). The algorithm was run for 100 iterations with non-fixed covariance Gaussian mixture model (GMM) clustering, and *k* = 7 modules. The selection of *k* = 7 was determined by the mean silhouette index per time point and overall BIC-corrected likelihood score (Additional file 1: Figure S3A, B). From ESCAROLE, we obtain a module assignment for each gene at each time point and identified sets of genes that transition in their module assignment across the eight time points (Fig. 2B).

We define transitioning gene sets from ESCAROLE results by grouping genes with a similar module transition profile with agglomerative hierarchical clustering (Fig. 3C, Additional file 1: Figure S3D). The pairwise distance between genes used for this clustering approach was the fraction of mismatches in the module assignment across the (8 point) time course. The distance threshold (to determine the cut on the dendrogram for the hierarchical clustering) and the minimum number of genes in a cluster were the input parameters to define the transitioning gene clusters in this approach. In choosing settings for these parameters, we tested different pairwise distance threshold values (those corresponding to 0-4 mismatches between module assignment profiles) and examined the resulting cluster sets for their size, overlap with differentially expressed genes, and enrichments of Gene Ontology (GO) and motif terms (see also the “Integrative analysis of RNA-seq and ATAC-seq time course data using the dynamic regulatory module networks algorithm” section). We chose a pairwise distance threshold of 0.26 in the hierarchical clustering analysis (corresponding to two mismatches across the 8-point time course) based on these results and used those clusters with 10 or more genes to define the 112 transitioning gene sets from the ESCAROLE results.

### Exploratory analysis of ATAC-seq data

#### Data pre-processing

Each of the eight ATAC-seq libraries was paired-end sequenced twice, and 54 to 235 million reads were obtained from each sequencing library (Additional file 1: Figure S4A and B). The data were aligned with Bowtie 2 [55] to the *M. truncatula* v5 genome, with 46–75% of the data found mappable (alignable) to the reference genome. Properly paired fragments with a quality score of 3 or greater were then obtained with “samtools view -Sb -q3 -f2,” (Properly paired, Additional file 1: Figure S4A, B) and duplicate-removal was applied with “samtools rmdup” [56] to define the final library data sets utilized (Selected, Additional file 1: Fig S4A, B).

Fragment length distributions of each time-point data set (Additional file 1: Figure S4C) present the expected ~10 bp DNA pitch but not nucleosome occupancy dependence first illustrated by Buenostro et al. [57]. This is consistent with previously published plant ATAC-seq data from Blajic et al. [58] (see Fig. 2A of that work). The absence of nucleosome occupancy dependence can be in part due to aspects of the ATAC-seq protocol implemented in plants versus mammals. Another explanation could be the large proportion of our reads mapping to promoter regions, which tend to be nucleosome depleted further explaining the diminished nucleosome pitch. Moreover, TSS-centric (±1kbp) distributions of selected fragments for each time point were analyzed using the ATACSeqQC [59] pipeline and the ChIPpeakAnno [60] toolset’s featureAlignedHeatmap function and found to be both favorable (Additional file 1: Figure S5A and B) and comparable to results from the Maher et al. *M. truncatula* data (Additional file 1: Figure S6A and B) analyzed in the same way.

Peak calling was performed by applying MACS2 [30] to ATAC-seq data from each time point using the command:

$$\textrm{macs}2\ \textrm{callpeak}-\textrm{t}<\textrm{bam}\ \textrm{file}>-\textrm{n}<\textrm{Name}>--\textrm{format}\ \textrm{BAMPE}-\textrm{gsize}=3.4\textrm{e}8$$

We mapped these peaks to genes if the center of a peak was within 10 kbp upstream and 1 kbp downstream of a gene transcription start site (TSS). Peaks called at each time point were merged across time points to generate a set of “universal peaks” using custom scripts [61] (Additional file 1: Figure S9A and B) based on two criteria: (1) peaks from two different time points had a Jaccard score overlap of 0.9 or higher, and (2) the peak from one time point was contained within the peak detected in another time point. FriP values of the peaks called in each time point were found to be favorable [62] (Additional file 1: Figure S9A), i.e., > 0.30 for all time points in accordance with ENCODE consortium standards for ATAC-seq peak-calling results. Annotations of the universal peak set (Fig. 3D, Additional file 1: Figure S9C and G) were generated in three steps: (1) annotating all peaks centered within 2 kbp upstream and 100 kbp downstream a gene TSS as “Promoter” peaks, (2) annotating any remaining peaks centered within 2–10 kbp upstream of a gene TSS as “Upstream”, and (3) using the results of the Homer annotatePeaks.pl tool [63] for all remaining peaks. The proportion of universal peaks mapped to “Promoter” regions under this definition is 32.1%.

#### Correlation and clustering analysis

To enable quantitative comparison of the chromatin accessibility profiles across time, we aggregated the ATAC-seq read counts in two sets of genomic regions: (1) gene promoter regions (defined as 2 kbp upstream to 2 kbp downstream of a given gene TSS) and (2) universal peaks described above using custom scripts [64]. Briefly, we first generated the per base pair (per-bp) coverage of fragments for each time point data set with Bedtools [65] using the command *bedtools genomecov <bam file> -bp -pc*. For each ± 2 kbp gene promoter region, the average coverage per-bp was estimated, and log-ratio transformed relative to the global genome-wide average per-bp coverage in the respective time point data set. The genome-wide average per-bp coverage was obtained by dividing the total coverage on any base pair by the length of the genome. The signals aggregated to the promoter were used for downstream principal component analysis (Fig. 3C). The Pearson correlation of aggregated promoter signals for replicate data sets was 0.965–0.990 across time-points (Additional file 1: Figure S7), indicating high consistency, also indicated by the similarity of PCA results for the replicate data sets (Additional file 1: Figure S8A).

The signal for the universal peaks was similarly quantified by the log-ratio of the mean per-bp coverage of the respective peak region relative to the global average per-bp coverage. For both data sets, this was followed by quantile normalization across time points, providing a continuous measure of the accessibility of gene promoter and peak regions.

To evaluate the relationship between gene expression and either promoter or universal peak accessibility, we first performed a zero-mean transformation of each gene’s expression profile and the corresponding accessibility profiles. Next, a Pearson’s correlation was estimated. To assess the significance of correlation, we generated a null distribution of correlations from 1000 random permutations of the time points. We computed a *P*-value that estimates the probability of observing a correlation in the permuted data more significant in magnitude than an observed correlation, treating positive and negative correlations separately. For the eight time points in this data set Pearson’s correlations were typically significant (*P* < = 0.05) when > 0.50 or < − 0.50. The zero-meaned promoter and universal peak accessibility profiles were clustered with k-means clustering, and the optimal settings for *k* were determined separately for each data set. In both cases, the silhouette index (computed with correlation distance metric) was used to select the optimal *k*. Here, *k* = 6 clusters were chosen for the promoter accessibility data (Fig. 3A, Additional file 1: Figure S8B). For the universal peak accessibility profile clusters, we additionally used enrichments for motifs within the clusters of peaks to determine the optimal setting of *k* = 9 clusters (Additional file 1: Figure S9D, E). The clusters were enriched for motif instances of several known regulators (Additional file 1: Figure S9F). Furthermore, the peaks in clusters 1 and 2 were more likely to be annotated as intergenic regions than peaks in any other cluster (Additional file 1: Figure S9G).

### Integrative analysis of RNA-seq and ATAC-seq time course data using the dynamic regulatory module networks algorithm

We applied a novel algorithm, dynamic regulatory module networks (DRMN) [18, 66, 67], to our RNA-seq and ATAC-seq time course data set to identify *cis*-regulatory elements and transcription factors associated with genes that exhibit dynamic behavior. The inputs to this algorithm are the RNA-seq time series data, the number of expression modules, and regulatory features for each time point derived from the ATAC-seq time course by examining the genomic region around a gene’s TSS. The algorithm outputs gene expression modules (states) for each time point and their associated regulatory programs comprising the *cis*-regulatory elements that best predict gene expression of a particular module.

To obtain the *cis*-regulatory features for each gene, we used 333 *M. truncatula* motif position weight matrices from the CisBP v1.02 database [33] and seven curated motifs of interest (including those for NIN, CYCLOPS (CYC-RE), and NSP1 and other binding motifs). ATAC-seq activity was aggregated for those known motif instances in the manner described above for promoter and universal peak regions. Motif finding was done for each of the associated position weight matrices using the *pwmmatch.exact.r* script (from the PIQ pipeline [68]) using the default log-likelihood score threshold of 5. Motifs mapped to 10 kbp upstream to 1 kbp downstream of gene TSSs were assigned as potential features describing the corresponding gene’s expression. This distance cutoff was motivated by the experimental validation of the *daphne* mutation for the *NIN (NODULE INCEPTION)* gene in *Lotus japonicus* by Yoro et al. [69], which is an insertion in a regulatory site ~7 kbp from this gene and affects its expression. Moreover, Liu et al. [70] have likewise validated similar regulatory interactions between sites ~5 kbp upstream of the *NIN* gene in *M. truncatula.* For each gene, the accessibility of multiple instances of the same motif mapped to that gene was summed. Finally, the aggregated motif accessibility feature data were merged across the time course and quantile normalized [64]. The normalized accessibility data for ±2 kbp promoter regions were also included as a predictive feature of gene expression.

The DRMN algorithm takes as input the number of modules, *k* and uses a regularized regression model, Fused Lasso [71], to learn regression models for each module, *k*, for all time points jointly. This has the following objective:

$$\underset{\Theta}{\min}\sum_c{\left\Vert {X}_{c,k}-{Y}_{c,k}{\Theta}_{c,k}^T\right\Vert}_2^2+{\rho}_1{\left\Vert {\Theta}_k\right\Vert}_1+\sum_{c,c^{\prime }}{\rho}_2{\left\Vert {\Theta}_{c,k}-{\Theta}_{c\prime, k}\right\Vert}_1+{\rho}_3{\left\Vert {\Theta}_k\right\Vert}_{2,1}$$

Here, *X*_*c*,*k*_ is the *n*_*k*_ *X* 1 vector of expression levels for *n*_*k*_ genes in modules *k* for time point *c*, *Y*_*c*, *k*_ *is n*_*k*_ X *p* motif-accessibility feature matrix corresponding to the same genes, ${\Theta}_{c,k}^T,$ are the regression coefficients which represent the quantified association of gene expression with individual regulatory motif features. Here, Θ_*k*_ is the matrix of coefficients across time points. The sum over *c*, *c*^′^ represents the sum over pairs of consecutive time points. Specifically, here, ‖.‖_1_ is the *l*_1_ norm (sum of absolute values), ‖.‖_2_ is the *l*_2_ norm (square-root of the sum of each value), and ‖.‖_2, 1_ is the *l*_1,2_ norm, i.e., the sum of the *l*_2_ norm of the columns of the given matrix. Furthermore, *ρ*_1_, *ρ*_2_, and *ρ*_3_ are hyper-parameters of the model that need to be tuned for optimal training and inference of DRMNs. These parameters represent (1) a sparsity penalty, (2) enforcing similarity of features for consecutive time points, and (3) enforcing an overall similarity of feature selection across all time points. We used several criteria to determine these hyper-parameter settings. The most important is the Pearson correlation of actual and predicted expression in threefold cross-validation settings to assess the resulting predictive power of models inferred for varied settings of the hyperparameters. Additionally, the quality of the clustering (silhouette index scores), the BIC-corrected likelihood score, and stability of predictive power in threefold cross-validation (Additional file 1: Figure S10A, C) were considered. We first varied *ρ*_1_ (values of 1, 5–60 in increments of 5, and 75 and 100) and *ρ*_2_ (values of 0–60 in increments of 5, and 75 and 100) independently and assessed the resulting predictive power for all models inferred. Predictive power generally monotonically decreased with increasing values of either parameter for values of *ρ*_1_>10, while for *ρ*_2_< = 25, the clustering was unstable. A choice was made for *ρ*_1_ = 5 over *ρ*_1_ = 1, since predictive power correlation was marginally higher for *ρ*_2_ = 30–60.

With the *ρ*_1_ parameter fixed to 5, a second independent scan of *ρ*_2_ and *ρ*_3_ was performed_,_ with (1) *ρ*_2_ varied from 25–60 in increments of 5, 75, and 100, and (2) *ρ*_3_ scanned for values of 0–60 in increments of 5, 75, and 100. For settings of *ρ*_3_ = 5–20, there tended to be unstable predictive power of the least expressed module, recovering comparable but not greater performance compared to results for *ρ*_3_ = 0 or *ρ*_3_ > 20, indicating no advantage for setting *ρ*_3_ > 0. We considered the cross-validation predictive power, silhouette index of modules, and similarity to ESCAROLE modules, in determining a setting for *ρ*_2_ (Additional file 1: Figure S10A). Comparable performance was found for *ρ*_2_ = 30–60, but *ρ*_2_ = 45 and 50 maximized the mean threefold cross-validation performance. We selected *ρ*_2_ = 45, as it was the lower of the two settings to avoid unnecessarily high values for a hyperparameter. Based on this assessment results for the hyperparameter settings of *ρ*_1_ = 5, *ρ*_2_ = 45, and *ρ*_3_ = 0 were chosen.

We ran DRMN on our time-course data set for *k* = 7 input modules, based on the optimal numbers of modules determined in the ESCAROLE analysis. Each module (Additional file 2: Table S1) was predicted to have multiple regulators based on DRMN’s fused regression model. To allow initial interpretation of the regulators, we filtered them as follows: (1) the magnitude of regulator-module edge-weights (Additional file 2: Table S2) in at least one time point being greater than 0.02 and (2) the regulatory motif being enriched in the module (FDR corrected *q*-value from hyper-geometric test, *q* < 0.05) for all time points (Additional file 1: Figure S11, Additional file 2: Table S3). The modules was also tested for enrichment of GO terms, using an FDR corrected hypergeometric test (*q* < 0.05) to define significant enrichment (Additional file 1: Figure S12, Additional file 2: Table S3).

To identify module network edges that were significantly varying in time we first merged module network edge weights across time points per module and identified those edge weights that were significantly varying (*t*-test *P* < 0.05 as implemented in MATLAB with the ttest2() function) across the 0–1 and 2–24 h portions of the time course. The choice to compare across the 1–> 2 h time point transition was motivated by the observation of module reorganization at this time window (Fig. 5C). Those regulatory edges found to be significantly varying are likely important at the module level of organization (Fig. 6).

To identify gene sets that transition in their expression due to changes in their predictive regulatory programs, we grouped genes that changed their DRMN inferred module assignment across time points using the same agglomerative hierarchical clustering approach applied in the ESCAROLE transitioning gene clustering analysis. We performed GO and motif enrichment on these gene sets as well to assess the optimal threshold for cutting the dendrogram (Additional file 1: Figure S13A). In total, we identified 79 gene sets spanning 10,176 genes. These gene sets were further analyzed using a regularized regression approach (described below) to identify regulators for each gene set.

#### Inferring fine-grained regulator-target interactions

We identified fine-grained regulator gene interactions by predicting regulators for individual genes in transitioning gene sets using a structured sparsity approach called multi-task group lasso (MTG-LASSO, Fig. 7A). MTG-LASSO is a type of multi-task learning framework, where one performs a regression for multiple tasks simultaneously to share information among the tasks. Here, each gene in the gene set is a task, and MTG-LASSO enables us to select the same regulator (motif) for all genes in the set but with different regression parameters. The regulator identity defines the “group” in MTG-LASSO which includes the regression weights for the regulator for all genes in the set. MTG-LASSO selects or unselects entire groups of regression weights. The MTG-LASSO objective for each gene set is:

$$\underset{\Theta}{\min}\sum_g\frac{1}{2}{\left\Vert {X}_g-\sum_m{Y}_{m,g}{\Theta}_{m,g}\right\Vert}_2^2+\uplambda {\left\Vert \Theta \right\Vert}_{1/2}$$

Here, *X*_*g*_ is the expression profile over time for gene *g*, and *Y*_*m*, *g*_ is the vector of motif accessibility features for motif *m* and gene *g* over time. The parameters Θ_*m*, *g*_ are the regression coefficients for predicting the expression of *g* using the feature data for motif *m*. This second term denotes the ‖.‖_1/2_ norm defined as $\sum_m\sum_g{\Theta}_{m,g}^2$ and is used for (1) penalizing the number selected motif features according to the *l*_1_ norm and (2) enforcing smoothness of the regression coefficients across genes according to the *l*_2_ norm. λ is the hyper-parameter for controlling the group structure.

For each of the 79 transitioning gene sets, MTG-LASSO was applied (using the SLEP v4.1 package [35] in MATLAB [72]) to infer the most predictive regulatory features of gene expression over time from the same motif accessibility features used in the DRMN analysis. For each gene set, we applied MTG-LASSO in a leave-one-out testing mode (Additional file 1: Figure S14), where each of the eight time points was left out one at a time, a model was fit on the remaining seven, and predictive power (Pearson's correlation) was computed on the left-out time point. For each regulator, we calculated a *P*-value to assess the significance of the frequency with which a given regulator was selected relative to random. This was achieved by randomizing the data 40 times and estimating a null distribution for the rate with which that regulator was selected across folds. A *Z*-test *P*-value was then obtained for the result relative to random.

We called a regulator significant if it was selected at least 6 of 8 time-point folds, and the number of times it was selected was significantly higher (*t* test *P* < 0.05) relative to random for the frequency of selection across folds. MTG-LASSO’s hyper-parameter, *λ*, was determined for each transitioning gene set from the range 0.20–0.99 (in intervals 0.10) based on (1) the mean Pearson’s correlation (predictive power) of the inferred regulatory features and (2) the number of regulators (5–15 for most gene sets) identified as significant such that the ratio of the number of identified regulators to number of target genes being close to 0.05 (Additional file 1: Figure S14). This approach identified 33 gene sets (of the original 79) with predicted regulators. For the remaining transitioning gene sets, significant regulators were not found either because the available predictive features were not good descriptions of the respective gene expression profiles or regulators were obtained for only one or two settings of *λ*, hindering an appropriate assessment of results.

For each of the 33 gene sets for which we identified regulators using MTG-LASSO (Additional file 1: Figure S14), we created regulator-target predictions between the significant regulatory features and member genes, defining 122,245 regulatory edges spanning 126 motifs for 5978 target genes (from 10,176 genes aggregated among the 79 transitioning gene clusters). Of the 126 motifs, we mapped 53 motifs to 278 *M. truncatula* regulator genes, including 31 well-studied regulators (specifically with common names in the v5 genome annotations). The remaining 73 motifs were assigned to 261 *M. truncatula* genes in the v5 genome assembly that were additionally identified as transcription factors (TFs). The relatively high number of motif to gene name mappings is because TF names were provided in CisBP v1.2 as systematic gene names from the v3/v3.5 *M. truncatula* genome assemblies rather than v5. We used a 70% BLAST similarity score to define mappings from *M. truncatula* v3/v3.5 genome systematic gene names to v5 genome systematic gene names.

#### Validation of predicted regulators of nodulation with RNAi

We used RNAi to validate three predicted regulators from our DRMN analysis, EIN3, ERF1, and IAA4-5. 104 bp region in the CDS specific to the gene of interest was amplified with 5′-CACC and inserted into pENTR™/D-TOPO® using directional TOPO® cloning and further recombined in vitro with the destination vector pK7GW1WG2(II)-RedRoot (https://gatewayvectors.vib.be/collection/pk7gwiwg2ii-redroot) using Gateway® LR Clonase® II enzyme mix using manufacturer’s instructions.

To validate RNAi, total RNA was extracted from transformed roots of each genotype using Qiagen RNeasy® Plant Mini kit and genomic DNA removed using TURBO DNA-free™ Kit (Ambion). First-strand cDNA was synthesized using RevertAid RT Reverse Transcription Kit (Thermo Scientific™). Quantitative RT-PCR was performed using BIORAD SsoAdvanced Universal SYBR Green Supermix on BIORAD CFX96™ Real-time system; C1000 Touch™ Thermal cycler. The *HEL* and *UBC9* genes were used as endogenous controls. Two (*EIN3*—*MtrunA17Chr5g0440591*) or three (*ERF1*—*MtrunA17Chr1g0186741*) technical replicates were used. A BLAST was performed for all primers against the *M. truncatula v5* genome to ensure specificity. The primers chosen for the validation of RNAi do not overlap with the RNAi regions (utilized primers provided in Additional file 1: Table S1).

The RNAi expression clones were introduced into *Agrobacterium rhizogenes* MSU440 with electroporation. Composite *M. truncatula* plants were generated as previously described [73]. Three weeks after transformation with *A. rhizogenes* MSU440, the roots were screened for red fluorescence of tdTomato, and the composite plants with red roots were transferred to growth pouches containing modified nodulation medium (MNM) [74]. The plants were acclimated for 4 days and inoculated with *S. meliloti* 1021 harboring pXLGD4 [75]. Two weeks post inoculation, live seedlings were stained for *lacZ* (5 mM potassium ferrocyanide, 5 mM potassium ferricyanide, and 0.08% X-gal in 0.1 M PIPES, pH 7) overnight at 37 °C. Roots were rinsed in distilled water, and nodules were visualized and counted under a Leica fluorescence stereomicroscope (Fig. 8B, Additional file 2: Table S5).

## Supplementary Information

**Additional file 1: Table S1.** Primers used in the RNAi validation study. **Figure S1.** Analysis workflow. **Figure S2.** Detailed DE gene statistics summary. **Figure S3.** Supplementary ESCAROLE clustering results. **Figure S4.** ATAC-seq data alignment statistics and fragment length distributions. **Figure S5.** ATAC-seq activity heatmaps and line plots for ±1 kb TSS regions in LCO-treatment data. **Figure S6.** ATAC-seq activity heatmaps and line plots for ±1 kb TSS regions in the comparable Maher et al. *Medicago* root sample data. **Figure S7.** Correlation of aggregated ATAC-seq activity for ±2 kb promoter regions. **Figure S8.** Supplementary ATAC-seq promoter analysis plots. **Figure S9.** Supplementary ATAC-seq peak-calling analysis plots. **Figure S10.** DRMN hyper-parameter tuning summary. **Figure S11.** DRMN module network edge-weight summary. **Figure S12.** DRMN module GO enrichment summary. **Figure S13.** Summary of ESCAROLE and DRMN transitioning gene set statistics and comparison. **Figure S14.** Summary of MTG-LASSO results and parameter tuning. **Figure S15.** Supplementary RNAi validation information.

**Additional file 2: Table S1.** DRMN module assignments (all genes, all time points). **Table S2.** Inferred module-network edge-weights from DRMN. **Table S3.** Module motif enrichments. **Table S4.** MTG-LASSO target predictions. **Table S5.** RNAi validation results.

## Data Availability

The datasets supporting the conclusions of this article are available in the GEO under accession GSE154845 [76]. All data generated or analyzed during this study are included in this published article, its supplementary information files, and publicly available repositories. Previously published data analyzed in support of this work consists of that of Larrainzar et al. [12] under NCBI BioProject accession PRJNA269201 [77] and of Maher et al. [28] under GEO accessions GSM2704259 and GSM2704260 [78]. The results from DRMN and MTG-LASSO gene target predictions are available in Additional file 2. A web-supplement for visualizing the MTG-LASSO results is available at https://medicago-drmnviz.discovery.wisc.edu.

## Abbreviations

ATAC-seq: Assay for Transposase-Accessible Chromatin using sequencing
CV: Cross-validation (appears in Figure S10)
DEGs: Differentially expressed genes
DRMN: Dynamic regulatory module networks—the primary algorithm and software tool utilized in this work
GMM: Gaussian mixture model
GO: Gene ontology
LASSO: Least absolute shrinkage and selection operator
MTG-LASSO: Multi-task group LASSO
PCA: Principal component analysis
RNA-seq: Ribonucleic acid sequencing
RNAi: Ribonucleic acid interference
TF: Transcription factor, i.e., regulatory protein
TPM: Transcripts per million
TSS: Transcription stop site
TTS: Transcription start site
