# Supplementary material for: Temporal change in chromatin accessibility predicts regulators of nodulation in Medicago truncatula
Source: BMC Biol. 2022 Nov 9;20:252. doi: 10.1186/s12915-022-01450-9 (PMC9647978; doi:10.1186/s12915-022-01450-9)
Supplement: Supplementary file 1 — Additional file 1: Table S1. Primers used in the RNAi validation study. Figure S1. Analysis workflow. Figure S2. Detailed DE gene statistics summary. Figure S3. Supplementary ESCAROLE clustering results. Figure S4. ATAC-seq data alignment statistics and fragment length distributions. Figure S5. ATAC-seq activity heatmaps and line plots for ±1 kb TSS regions in LCO-treatment data. Figure S6. ATAC-seq activity heatmaps and line plots for ±1 kb TSS regions in the comparable Maher et al. Medicago root sample data. Figure S7. Correlation of aggregated ATAC-seq activity for ±2 kb promoter regions. Figure S8. Supplementary ATAC-seq promoter analysis plots. Figure S9. Supplementary ATAC-seq peak-calling analysis plots. Figure S10. DRMN hyper-parameter tuning summary. Figure S11. DRMN module network edge-weight summary. Figure S12. DRMN module GO enrichment summary. Figure S13. Summary of ESCAROLE and DRMN transitioning gene set statistics and comparison. Figure S14. Summary of MTG-LASSO results and parameter tuning. Figure S15. Supplementary RNAi validation information. [file 12915_2022_1450_MOESM1_ESM.pdf]

This file includes:
Supplementary Table 1
Supplementary Figures 1-15

Table S1. Primers used in the RNAi validation study.

Purpose	Forward primer (5’-3’)	Reverse primer (5’-3’)
MtrunA17Chr1g0166011 RNAi	**CACC**ATGGAATTCAAGGCAACTGAGCT	GTCTTTTGTTATTCTTAACAACACTACC
MtrunA17Chr5g0440591 RNAi	**CACC**ATGATGATGTTTGAGGACATGGGG	CAGGCTCGGTTTGCCTGACAGAAGAAA
MtrunA17Chr1g0186741 RNAi	**CACC**ATGGATTCAAGTTCAACCTCAA	CGTTCTCGTTGAAAGGAAGATAGTTG
Validation of *MtrunA17Chr5g0440591* RNAi	TAACGCAATCCCAGGAAAGAA	GCTGATAAGAGAGAACCCAAGG
Validation of *MtrunA17Chr1g0186741* RNAi	GAACGAACCAAGGAAGGAGAA	CCATTCCTCGTCGAATCTCTTATC
Endogenous control (*HEL)*	AGGACGCATGAGCTTTTCAA	AGCAGAGACCAGCATAACAAT
Endogenous control (*UBC9)*	AGCAGTGGTCTCATACTTGGA C	AGCCCCGCTTTGACAATATC

SUPPLEMENTARY FIGURES 1-15

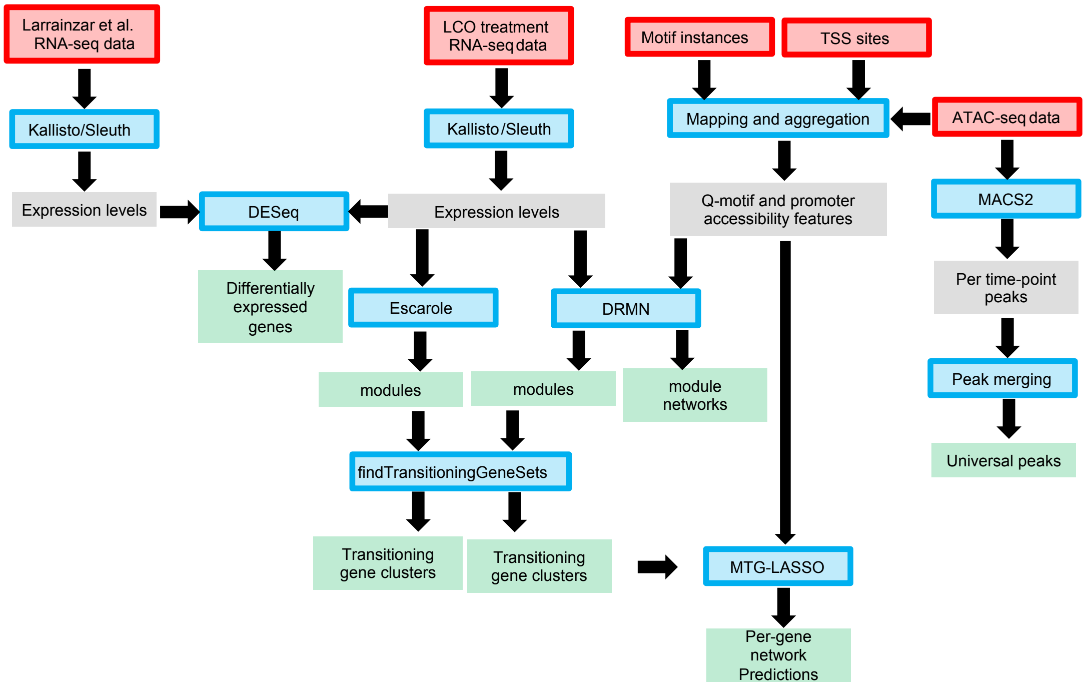

Figure S1. Flow chart of the data analysis. The input data are in red and the applied analysis algorithms and/or methods are in blue. Intermmediate and final analysis results are in gray and green, respectively.

A Present LCO treatment experiment

Time	Down	All	Up
0.25 hr	55	160	105
0.5 hr	793	1,633	840
1 hr	2,094	3,754	1,660
2 hr	2,574	4,297	1,723
4 hr	3,243	5,509	2,266
8 hr	4,235	8,049	3,814
24 hr	3,609	7,010	3,401
Total	7,052	12,839	7,540
All pairs	11,652	17,391	12,112

B

	Control	15 min	30 min	1 hr	2 hr	4 hr	8 hr
15 min	160						
30 min	1,633	406					
1 hr	3,754	1,612	352				
2 hr	4,297	3,816	3,219	2,787			
4 hr	5,509	6,124	6,621	7,073	3,687		
8 hr	8,049	8,605	9,043	9,550	6,678	1,327	
24 hr	7,010	7,888	8,305	9,208	7,296	4,651	4,050

C

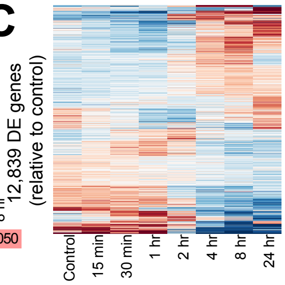

D

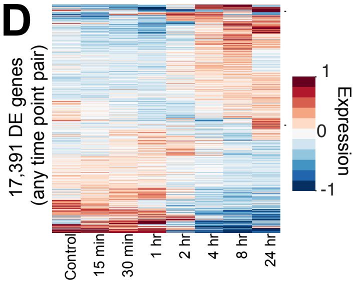

E Larrainzar et al. rhizobia treatment experiments

	DE genes statistics (relative to control)											
	WT (A17)			*nfp*			*lyk3*			*skl*		
Time	Down	All	Up	Down	All	Up	Down	All	Up	Down	All	Up
0.5 hr	186	1,033	847	18	200	182	30	420	390	15	289	274
1 hr	399	1,247	848	11	107	96	34	316	282	57	410	353
3 hr	489	1,045	556	168	365	197	267	563	296	226	497	271
6 hr	764	1,595	831	311	551	240	485	1,031	546	609	1,373	764
12 hr	536	1,372	836	321	720	399	414	938	524	967	2,185	1,218
24 hr	166	751	585	0	7	7	29	352	323	2,314	5,245	2,931
36 hr	632	1,525	893	261	532	271	448	1,025	577	3,888	7,326	3,438
48 hr	185	786	601	11	126	115	34	375	341	4,650	8,895	4,245
Total	1,808	4,576	2,873	539	1,460	935	912	2,407	1,517	5,689	10,891	5,425
All pairs	6,090	8,987	6,315	1,852	2,925	1,973	3,015	4,513	2,988	8,984	15,252	7,925

	DE gene statistics (relative to WT)								
Time	Down	All	Up	Down	All	Up	Down	All	Up
0.0 hr	23	33	10	10	25	15	67	142	75
0.5 hr	36	101	65	43	106	63	399	907	508
1 hr	110	248	138	143	203	60	296	715	419
3 hr	43	82	39	22	49	27	167	289	122
6 hr	91	474	383	17	47	30	334	725	391
12 hr	78	398	320	11	44	33	877	1,685	808
24 hr	89	311	222	26	82	56	1,834	3,455	1,621
36 hr	88	248	160	53	152	99	3,765	7,483	3,718
48 hr	182	370	188	65	201	136	3,959	8,017	4,058
Total	392	1,131	756	222	475	255	5,088	10,242	5,253

F

Comparison for down-regulated DE Genes

	# DE genes	LCO	WT	*nfp*	*lyk3*	*skl*	*nfp* vs. WT	*lyk3* vs. WT
LCO-treatment	7,052							
WT	1,808	0.22						
nfp	539	0.11	0.37					
lyk3	912	0.15	0.52	0.55				
skl	5,689	0.39	0.36	0.15	0.22			
nfp vs. WT	392	0.04	0.08	0.04	0.05	0.04		
lyk3 vs. WT	222	0.01	0.03	0.01	0.03	0.02	0.15	
skl vs. WT	5,088	0.13	0.08	0.03	0.04	0.05	0.05	0.03

Comparison for all DE genes

	# DE genes	LCO	WT	*nfp*	*lyk3*	*skl*	*nfp* vs. WT	*lyk3* vs. WT
LCO-treatment	12,839							
WT	4,576	0.40						
nfp	1,460	0.17	0.37					
lyk3	2,407	0.26	0.56	0.51				
skl	10,891	0.53	0.43	0.20	0.29			
nfp vs. WT	1,131	0.10	0.27	0.15	0.26	0.13		
lyk3 vs. WT	475	0.04	0.12	0.05	0.10	0.05	0.28	
skl vs. WT	10,242	0.48	0.37	0.15	0.22	0.72	0.15	0.06

Comparison for up-regulated DE genes

	# DE genes	LCO	WT	*nfp*	*lyk3*	*skl*	*nfp* vs. WT	*lyk3* vs. WT
LCO-treatment	7,540							
WT	2,873	0.25						
nfp	935	0.13	0.35					
lyk3	1,517	0.18	0.57	0.48				
skl	5,425	0.33	0.39	0.20	0.29			
nfp vs. WT	756	0.08	0.32	0.12	0.31	0.16		
lyk3 vs. WT	255	0.02	0.12	0.05	0.07	0.06	0.30	
skl vs. WT	5,253	0.27	0.24	0.10	0.15	0.08	0.10	0.03

F-score

G

Comparison for down-regulated DE Genes

Comparison with LCO-treatment data result	Gene Stats	t=0-15 min	t=0-30 min	t=0-1 hr	t=0-2hr	t=0-4 hr	t=0-8 hr	t=0-24 hr
	WT (A17)							
Gene stats		55	793	2,094	2,574	3,243	4,235	3,609
t=0-30 min	186	0.03	0.06	0.05	0.02	0.02	0.02	0.02
t=0-1 hr	399	0.02	0.11	0.09	0.04	0.03	0.03	0.03
t=0-3 hr	489	0.04	0.15	0.16	0.10	0.10	0.08	0.03
t=0-6 hr	764	0.02	0.16	0.18	0.12	0.13	0.13	0.06
t=0-12 hr	536	0.01	0.10	0.09	0.07	0.10	0.13	0.10
t=0-24 hr	166	0.01	0.02	0.02	0.02	0.02	0.01	0.02
t=0-36 hr	632	0.01	0.13	0.12	0.09	0.12	0.13	0.09
t=0-48 hr	185	0.01	0.03	0.03	0.02	0.02	0.01	0.02
	nfp							
Gene stats		55	793	2,094	2,574	3,243	4,235	3,609
t=0-30 min	18	0.03	0.01	0.01	0.00	0.00	0.00	0.00
t=0-1 hr	11	0.03	0.00	0.00	0.00	0.00	0.00	0.00
t=0-3 hr	168	0.03	0.08	0.07	0.04	0.05	0.04	0.02
t=0-6 hr	311	0.03	0.11	0.11	0.06	0.09	0.09	0.04
t=0-12 hr	321	0.02	0.08	0.08	0.06	0.09	0.10	0.08
t=0-24 hr	0	0.00	0.00	0.00	0.00	0.00	0.00	0.00
t=0-36 hr	261	0.02	0.08	0.07	0.05	0.09	0.09	0.06
t=0-48 hr	11	0.00	0.00	0.00	0.00	0.00	0.00	0.00
	lyk3							
Gene stats		55	793	2,094	2,574	3,243	4,235	3,609
t=0-30 min	30	0.05	0.01	0.01	0.00	0.00	0.00	0.00
t=0-1 hr	34	0.02	0.01	0.01	0.00	0.00	0.00	0.00
t=0-3 hr	267	0.03	0.12	0.11	0.06	0.07	0.06	0.02
t=0-6 hr	485	0.02	0.15	0.16	0.09	0.11	0.11	0.04
t=0-12 hr	414	0.01	0.11	0.10	0.06	0.10	0.12	0.08
t=0-24 hr	29	0.00	0.00	0.00	0.00	0.00	0.00	0.00
t=0-36 hr	448	0.02	0.12	0.10	0.07	0.11	0.12	0.08
t=0-48 hr	34	0.02	0.00	0.00	0.00	0.00	0.00	0.01
	skl							
Gene stats		55	793	2,094	2,574	3,243	4,235	3,609
t=0-30 min	15	0.00	0.00	0.00	0.00	0.00	0.00	0.00
t=0-1 hr	57	0.00	0.03	0.02	0.01	0.01	0.01	0.00
t=0-3 hr	226	0.02	0.09	0.09	0.06	0.06	0.05	0.02
t=0-6 hr	609	0.02	0.15	0.17	0.11	0.13	0.12	0.06
t=0-12 hr	967	0.01	0.11	0.13	0.12	0.19	0.21	0.18
t=0-24 hr	2,314	0.01	0.11	0.16	0.16	0.19	0.20	0.22
t=0-36 hr	3,888	0.00	0.12	0.19	0.22	0.27	0.31	0.27
t=0-48 hr	4,650	0.01	0.11	0.19	0.21	0.23	0.25	0.24

Comparisons for all DE genes

Comparison with LCO-treatment data result	Gene Stats	t=0-15 min	t=0-30 min	t=0-1 hr	t=0-2hr	t=0-4 hr	t=0-8 hr	t=0-24 hr
	WT (A17)							
Gene stats		160	1,633	3,754	4,297	5,509	8,049	7,010
t=0-30 min	1,033	0.05	0.22	0.18	0.15	0.18	0.14	0.14
t=0-1 hr	1,247	0.05	0.21	0.20	0.18	0.18	0.16	0.16
t=0-3 hr	1,045	0.04	0.15	0.17	0.16	0.16	0.13	0.13
t=0-6 hr	1,595	0.04	0.17	0.20	0.21	0.22	0.18	0.17
t=0-12 hr	1,372	0.04	0.15	0.16	0.16	0.17	0.16	0.16
t=0-24 hr	751	0.04	0.08	0.08	0.08	0.09	0.07	0.08
t=0-36 hr	1,525	0.03	0.14	0.16	0.16	0.17	0.15	0.16
t=0-48 hr	786	0.03	0.06	0.07	0.08	0.08	0.06	0.08
	nfp							
Gene stats		160	1,633	3,754	4,297	5,509	8,049	7,010
t=0-30 min	200	0.03	0.07	0.04	0.04	0.04	0.03	0.03
t=0-1 hr	107	0.06	0.04	0.02	0.03	0.03	0.02	0.02
t=0-3 hr	365	0.04	0.08	0.08	0.07	0.07	0.06	0.05
t=0-6 hr	551	0.03	0.09	0.10	0.09	0.11	0.09	0.07
t=0-12 hr	720	0.04	0.09	0.11	0.10	0.10	0.10	0.11
t=0-24 hr	7	0.01	0.00	0.00	0.00	0.00	0.00	0.00
t=0-36 hr	532	0.03	0.08	0.08	0.07	0.08	0.08	0.08
t=0-48 hr	126	0.03	0.02	0.01	0.02	0.02	0.01	0.02
	lyk3							
Gene stats		160	1,633	3,754	4,297	5,509	8,049	7,010
t=0-30 min	420	0.07	0.14	0.09	0.07	0.08	0.07	0.07
t=0-1 hr	316	0.06	0.09	0.07	0.06	0.07	0.06	0.06
t=0-3 hr	563	0.05	0.11	0.11	0.10	0.10	0.08	0.08
t=0-6 hr	1,031	0.04	0.15	0.16	0.16	0.16	0.13	0.13
t=0-12 hr	938	0.04	0.13	0.14	0.12	0.14	0.13	0.12
t=0-24 hr	352	0.05	0.05	0.04	0.05	0.05	0.03	0.04
t=0-36 hr	1,025	0.04	0.12	0.13	0.13	0.14	0.12	0.12
t=0-48 hr	375	0.05	0.05	0.04	0.05	0.05	0.04	0.05
	skl							
Gene stats		160	1,633	3,754	4,297	5,509	8,049	7,010
t=0-30 min	289	0.09	0.12	0.08	0.06	0.06	0.05	0.05
t=0-1 hr	410	0.07	0.12	0.10	0.09	0.09	0.07	0.07
t=0-3 hr	497	0.05	0.11	0.11	0.09	0.10	0.07	0.07
t=0-6 hr	1,373	0.04	0.15	0.18	0.18	0.20	0.16	0.15
t=0-12 hr	2,185	0.04	0.14	0.19	0.19	0.22	0.22	0.21
t=0-24 hr	5,245	0.02	0.12	0.20	0.22	0.24	0.28	0.29
t=0-36 hr	7,326	0.02	0.15	0.25	0.27	0.31	0.36	0.35
t=0-48 hr	8,895	0.01	0.13	0.24	0.27	0.29	0.35	0.35

Comparison for up-regulated DE genes

Comparison with LCO-treatment data result	Gene Stats	t=0-15 min	t=0-30 min	t=0-1 hr	t=0-2hr	t=0-4 hr	t=0-8 hr	t=0-24 hr
	WT (A17)							
Gene stats		105	840	1,660	1,723	2,266	3,814	3,401
t=0-30 min	847	0.04	0.21	0.17	0.04	0.02	0.02	0.03
t=0-1 hr	848	0.04	0.18	0.16	0.05	0.03	0.02	0.03
t=0-3 hr	556	0.04	0.10	0.12	0.15	0.15	0.09	0.06
t=0-6 hr	831	0.05	0.13	0.15	0.17	0.20	0.14	0.08
t=0-12 hr	836	0.04	0.12	0.11	0.13	0.16	0.14	0.12
t=0-24 hr	585	0.05	0.09	0.09	0.10	0.11	0.08	0.09
t=0-36 hr	893	0.03	0.08	0.08	0.11	0.13	0.11	0.12
t=0-48 hr	601	0.04	0.06	0.07	0.08	0.08	0.06	0.09
	nfp							
Gene stats		105	840	1,660	1,723	2,266	3,814	3,401
t=0-30 min	182	0.03	0.08	0.04	0.01	0.00	0.00	0.01
t=0-1 hr	96	0.06	0.03	0.02	0.01	0.00	0.00	0.00
t=0-3 hr	197	0.03	0.05	0.06	0.07	0.08	0.05	0.03
t=0-6 hr	240	0.01	0.03	0.04	0.06	0.10	0.08	0.04
t=0-12 hr	399	0.00	0.01	0.02	0.03	0.07	0.08	0.11
t=0-24 hr	7	0.00	0.00	0.00	0.00	0.00	0.00	0.00
t=0-36 hr	271	0.02	0.02	0.02	0.03	0.06	0.06	0.07
t=0-48 hr	115	0.03	0.03	0.02	0.03	0.04	0.02	0.03
	lyk3							
Gene stats		105	840	1,660	1,723	2,266	3,814	3,401
t=0-30 min	390	0.06	0.17	0.12	0.02	0.01	0.01	0.02
t=0-1 hr	282	0.06	0.10	0.08	0.02	0.01	0.01	0.01
t=0-3 hr	296	0.05	0.08	0.10	0.10	0.10	0.05	0.03
t=0-6 hr	546	0.04	0.10	0.11	0.14	0.16	0.11	0.07
t=0-12 hr	524	0.04	0.09	0.09	0.11	0.15	0.12	0.11
t=0-24 hr	323	0.05	0.07	0.06	0.07	0.09	0.05	0.06
t=0-36 hr	577	0.04	0.07	0.07	0.08	0.12	0.09	0.11
t=0-48 hr	341	0.05	0.06	0.06	0.06	0.08	0.05	0.08
	skl							
Gene stats		105	840	1,660	1,723	2,266	3,814	3,401
t=0-30 min	274	0.05	0.13	0.08	0.01	0.01	0.00	0.01
t=0-1 hr	353	0.03	0.09	0.07	0.02	0.02	0.01	0.02
t=0-3 hr	271	0.06	0.09	0.10	0.10	0.10	0.05	0.03
t=0-6 hr	764	0.04	0.11	0.11	0.18	0.23	0.16	0.11
t=0-12 hr	1,218	0.03	0.08	0.09	0.11	0.18	0.17	0.18
t=0-24 hr	2,931	0.01	0.06	0.08	0.08	0.14	0.20	0.22
t=0-36 hr	3,438	0.01	0.06	0.09	0.09	0.18	0.26	0.24
t=0-48 hr	4,245	0.01	0.05	0.09	0.08	0.15	0.22	0.22

Figure S2. Analysis of differentially expressed (DE) genes. (A) Summary statistics (red) of DE genes identified with DESeq (adjusted $P<0.05$) in the LCO-treatment data set compared to the control (0 min) time point. Total indicates the size of the union set identified across all time points. All pairs is the size of the union of DE genes identified between any pair of time points (See **B**). (**B**) Numbers of DE genes called between all pairs of time points in the LCO dataset. (**C**) Expression heat-maps of DE genes called in the LCO dataset relative to control. Shown are the log-zero-mean transformed data for each gene. (**D**) Expression heatmaps of DE genes called between all time point pairs. (**E**) Statistics of DE genes called from the four rhizobial-treatment time course from Larrainzar et al. data sets (WT, *nfp*, *lyk3*, and *skl*). Statistics are shown for all and specifically up or down regulated DE genes (relative to control or WT conditions). The Larrainzar et al. data results are shown for all time points both relative to control (within all four experiments) and relative to WT (for the three mutant strains). (**F**) The overlap (F-score, see **Methods,** blue color scale) of DE gene sets from our LCO-treatment dataset and the Larrainzar et al. data sets across all time-points relative to control. **(G)** Per-time-point DE gene set overlaps between the LCO and Larrainzar et al. datasets. The *skl/ein2* mutant data DE gene set is the most similar to that of our dataset, indicating LCO response is predominant in both experiments.

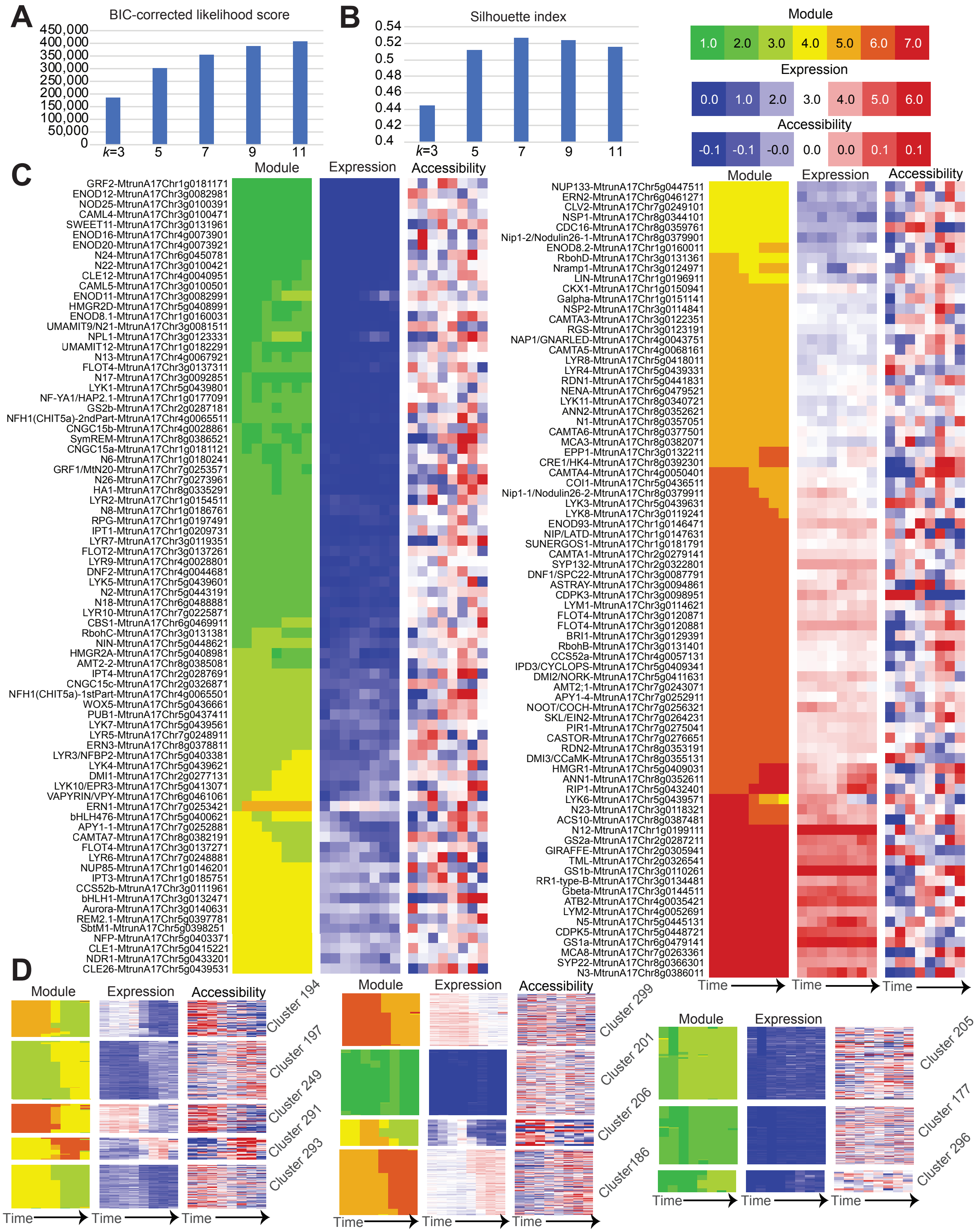

A
BIC-corrected likelihood score
450,000
400,000
350,000
300,000
250,000
200,000
150,000
100,000
50,000
0
k=3
5
7
9
11
B
Silhouette index
0.54
0.52
0.5
0.48
0.46
0.44
0.42
0.4
k=3
5
7
9
11
Module
1.0
2.0
3.0
4.0
5.0
6.0
7.0
Expression
0.0
1.0
2.0
3.0
4.0
5.0
6.0
Accessibility
-0.1
-0.1
-0.0
0.0
0.0
0.1
0.1
C
Module
Expression
Accessibility
GRF2-MtrunA17Chr1g0181171
ENOD12-MtrunA17Chr3g0082981
NOD25-MtrunA17Chr3g0100391
CAML4-MtrunA17Chr3g0100471
SWEET11-MtrunA17Chr3g0131961
ENOD16-MtrunA17Chr4g0073901
ENOD20-MtrunA17Chr4g0073921
N24-MtrunA17Chr6g0450781
N22-MtrunA17Chr3g0100421
CLE12-MtrunA17Chr4g0040951
CAML5-MtrunA17Chr3g0100501
ENOD11-MtrunA17Chr3g0082991
HMGR2D-MtrunA17Chr5g0408991
ENOD8.1-MtrunA17Chr1g0160031
UMAMIT9/N21-MtrunA17Chr3g0081511
NPL1-MtrunA17Chr3g0123331
UMAMIT12-MtrunA17Chr1g0182291
N13-MtrunA17Chr4g0067921
FLOT4-MtrunA17Chr3g0137311
N17-MtrunA17Chr3g0092851
LYK1-MtrunA17Chr5g0439801
NF-YA1/HAP2.1-MtrunA17Chr1g0177091
GS2b-MtrunA17Chr2g0287181
NFH1(CHIT5a)-2ndPart-MtrunA17Chr4g0065511
CNGC15b-MtrunA17Chr4g0028861
SymREM-MtrunA17Chr8g0386521
CNGC15a-MtrunA17Chr1g0181121
N6-MtrunA17Chr1g0180241
GRF1/MtN20-MtrunA17Chr7g0253571
N26-MtrunA17Chr7g0273961
HA1-MtrunA17Chr8g0335291
LYR2-MtrunA17Chr1g0154511
N8-MtrunA17Chr1g0186761
RPG-MtrunA17Chr1g0197491
IPT1-MtrunA17Chr1g0209731
LYR7-MtrunA17Chr3g0119351
FLOT2-MtrunA17Chr3g0137261
LYR9-MtrunA17Chr4g0028801
DNF2-MtrunA17Chr4g0044681
LYK5-MtrunA17Chr5g0439601
N2-MtrunA17Chr5g0443191
N18-MtrunA17Chr6g0488881
LYR10-MtrunA17Chr7g0225871
CBS1-MtrunA17Chr6g0469911
RbohC-MtrunA17Chr3g0131381
NIN-MtrunA17Chr5g0448621
HMGR2A-MtrunA17Chr5g0408981
AMT2-2-MtrunA17Chr8g0385081
IPT4-MtrunA17Chr2g0287691
CNGC15c-MtrunA17Chr2g0326871
NFH1(CHIT5a)-1stPart-MtrunA17Chr4g0065501
WOX5-MtrunA17Chr5g0436661
PUB1-MtrunA17Chr5g0437411
LYK7-MtrunA17Chr5g0439561
LYR5-MtrunA17Chr7g0248911
ERN3-MtrunA17Chr8g0378811
LYR3/NFBP2-MtrunA17Chr5g0403381
LYK4-MtrunA17Chr5g0439621
DMI1-MtrunA17Chr2g0277131
LYK10/EPR3-MtrunA17Chr5g0413071
VAPYRIN/VPY-MtrunA17Chr6g0461061
ERN1-MtrunA17Chr7g0253421
bHLH476-MtrunA17Chr5g0400621
APY1-1-MtrunA17Chr7g0252881
CAMTA7-MtrunA17Chr8g0382191
FLOT4-MtrunA17Chr3g0137271
LYR6-MtrunA17Chr7g0248881
NUP85-MtrunA17Chr1g0146201
IPT3-MtrunA17Chr1g0185751
CCS52b-MtrunA17Chr3g0111961
bHLH1-MtrunA17Chr3g0132471
Aurora-MtrunA17Chr3g0140631
REM2.1-MtrunA17Chr5g0397781
SbtM1-MtrunA17Chr5g0398251
NFP-MtrunA17Chr5g0403371
CLE1-MtrunA17Chr5g0415221
NDR1-MtrunA17Chr5g0433201
CLE26-MtrunA17Chr5g0439531
Module
Expression
Accessibility
NUP133-MtrunA17Chr5g0447511
ERN2-MtrunA17Chr6g0461271
CLV2-MtrunA17Chr7g0249101
NSP1-MtrunA17Chr8g0344101
CDC16-MtrunA17Chr8g0359761
Nip1-2/Nodulin26-1-MtrunA17Chr8g0379901
ENOD8.2-MtrunA17Chr1g0160011
RbohD-MtrunA17Chr3g0131361
Nramp1-MtrunA17Chr3g0124971
LIN-MtrunA17Chr1g0196911
CKX1-MtrunA17Chr1g0150941
Galpha-MtrunA17Chr1g0151141
NSP2-MtrunA17Chr3g0114841
CAMTA3-MtrunA17Chr3g0122351
RGS-MtrunA17Chr3g0123191
NAP1/GNARLED-MtrunA17Chr4g0043751
CAMTA5-MtrunA17Chr4g0068161
LYR8-MtrunA17Chr5g0418011
LYR4-MtrunA17Chr5g0439331
RDN1-MtrunA17Chr5g0441831
NENA-MtrunA17Chr6g0479521
LYK11-MtrunA17Chr8g0340721
ANN2-MtrunA17Chr8g0352621
N1-MtrunA17Chr8g0357051
CAMTA6-MtrunA17Chr8g0377501
MCA3-MtrunA17Chr8g0382071
EPP1-MtrunA17Chr3g0132211
CRE1/HK4-MtrunA17Chr8g0392301
CAMTA4-MtrunA17Chr4g0050401
COI1-MtrunA17Chr5g0436511
Nip1-1/Nodulin26-2-MtrunA17Chr8g0379911
LYK3-MtrunA17Chr5g0439631
LYK8-MtrunA17Chr3g0119241
ENOD93-MtrunA17Chr1g0146471
NIP/LATD-MtrunA17Chr1g0147631
SUNERGOS1-MtrunA17Chr1g0181791
CAMTA1-MtrunA17Chr2g0279141
SYP132-MtrunA17Chr2g0322801
DNF1/SPC22-MtrunA17Chr3g0087791
ASTRAY-MtrunA17Chr3g0094861
CDPK3-MtrunA17Chr3g0098951
LYM1-MtrunA17Chr3g0114621
FLOT4-MtrunA17Chr3g0120871
FLOT4-MtrunA17Chr3g0120881
BRI1-MtrunA17Chr3g0129391
RbohB-MtrunA17Chr3g0131401
CCS52a-MtrunA17Chr4g0057131
IPD3/CYCLOPS-MtrunA17Chr5g0409341
DMI2/NORK-MtrunA17Chr5g0411631
AMT2;1-MtrunA17Chr7g0243071
APY1-4-MtrunA17Chr7g0252911
NOOT/COCH-MtrunA17Chr7g0256321
SKL/EIN2-MtrunA17Chr7g0264231
PIR1-MtrunA17Chr7g0275041
CASTOR-MtrunA17Chr7g0276651
RDN2-MtrunA17Chr8g0353191
DMI3/CCaMK-MtrunA17Chr8g0355131
HMGR1-MtrunA17Chr5g0409031
ANN1-MtrunA17Chr8g0352611
RIP1-MtrunA17Chr5g0432401
LYK6-MtrunA17Chr5g0439571
N23-MtrunA17Chr3g0118321
ACS10-MtrunA17Chr8g0387481
N12-MtrunA17Chr1g0199111
GS2a-MtrunA17Chr2g0287211
GIRAFFE-MtrunA17Chr2g0305941
TML-MtrunA17Chr2g0326541
GS1b-MtrunA17Chr3g0110261
RR1-type-B-MtrunA17Chr3g0134481
Gbeta-MtrunA17Chr3g0144511
ATB2-MtrunA17Chr4g0035421
LYM2-MtrunA17Chr4g0052691
N5-MtrunA17Chr5g0445131
CDPK5-MtrunA17Chr5g0448721
GS1a-MtrunA17Chr6g0479141
MCA8-MtrunA17Chr7g0263361
SYP22-MtrunA17Chr8g0366301
N3-MtrunA17Chr8g0386011
Time
Time
Time
D
Module
Expression
Accessibility
Cluster 194
Cluster 197
Cluster 249
Cluster 291
Cluster 293
Time
Time
Time
Module
Expression
Accessibility
Cluster 299
Cluster 201
Cluster 206
Cluster186
Time
Time
Time
Module
Expression
Cluster 205
Cluster 177
Cluster 296
Time
Time
Time

Figure S3. ESCAROLE analysis of RNAseq data. (A-B) BIC-corrected likelihood scores **(A)** Silhouette index **(B)** for ESCAROLE results for varying settings of *k*, motivating *k*=7 as an appropriate choice for our analysis. **(C)** ESCAROLE module assignments, expression levels and row-zero-meaned promoter accessibility profiles for 152 genes of interest in root nodulation and N-fixing symbiosis (see legend, upper right-hand corner). **(D)** Example transitioning gene clusters identified with ESCAROLE module assignments. Shown are the module assignments, gene expression profiles, as well as promoter accessibility profiles (Same legend as in **(C)**). The specific gene set is numbered on the right.

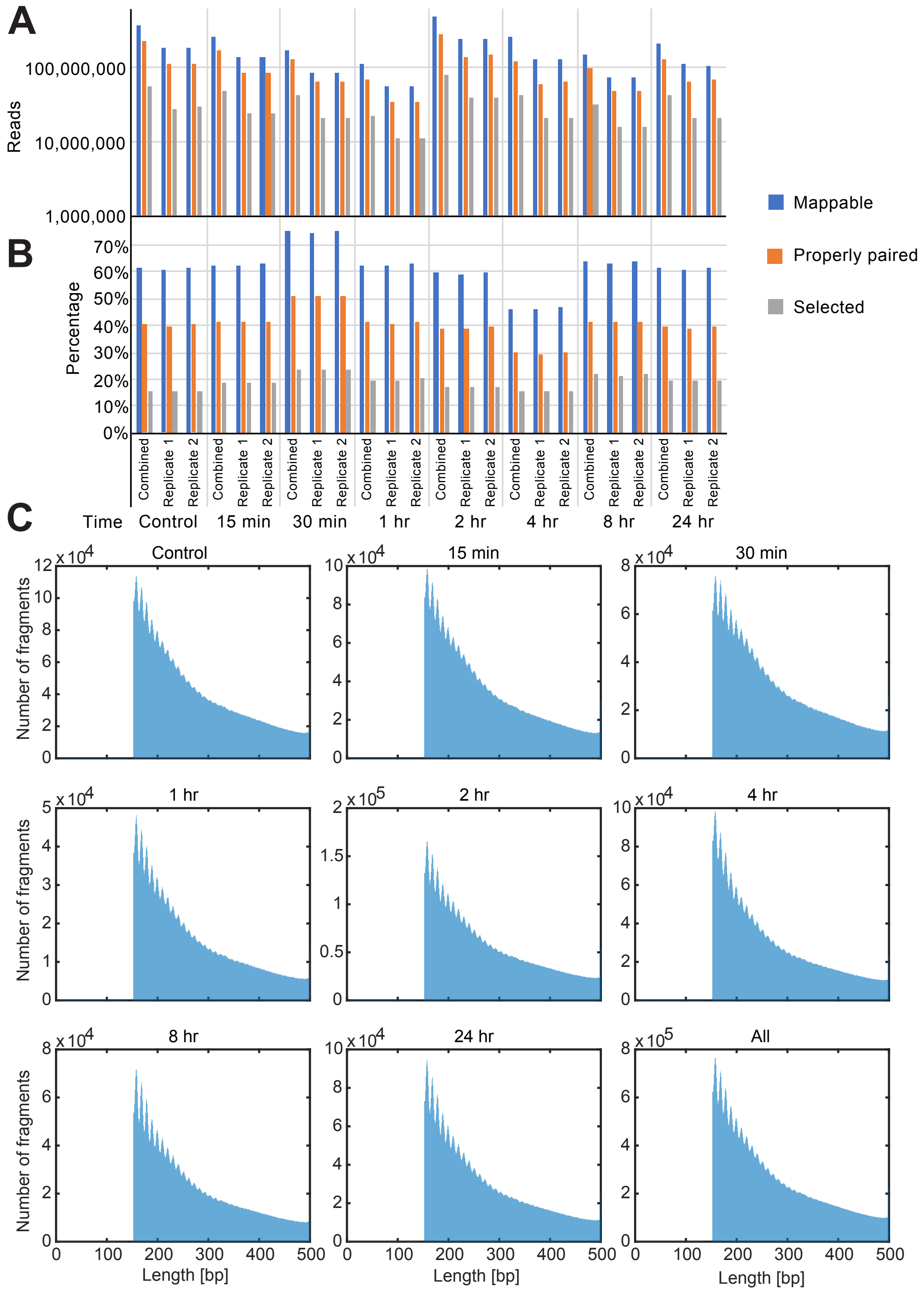
A
Reads
100,000,000
10,000,000
1,000,000
B
Percentage
70%
60%
50%
40%
30%
20%
10%
0%
Mappable
Properly paired
Selected
Combined
Replicate 1
Replicate 2
Time
Control
15 min
30 min
1 hr
2 hr
4 hr
8 hr
24 hr
C
Control
15 min
30 min
1 hr
2 hr
4 hr
8 hr
24 hr
All
Number of fragments
Length [bp]
x10^4
x10^5

Figure S4. Summary of ATAC-seq data alignment. (A) Read alignment statistics and **(B)** percentages of the data that are 1) mappable to the *M. truncatula* genome, 2) properly paired and 3) selected across replicates and time-points. The “selected” alignments are those meeting a mapping quality condition, and duplicate removal as described in the **Methods**. **(C)** Paired-end fragment length distributions shown for each time-point data set (and overall). Although nucleosome occupancy dependence is not prominent in these distributions, due to the specifics of the protocol implemented in this (plant) experiment, the ~10 bp DNA pitch is present.

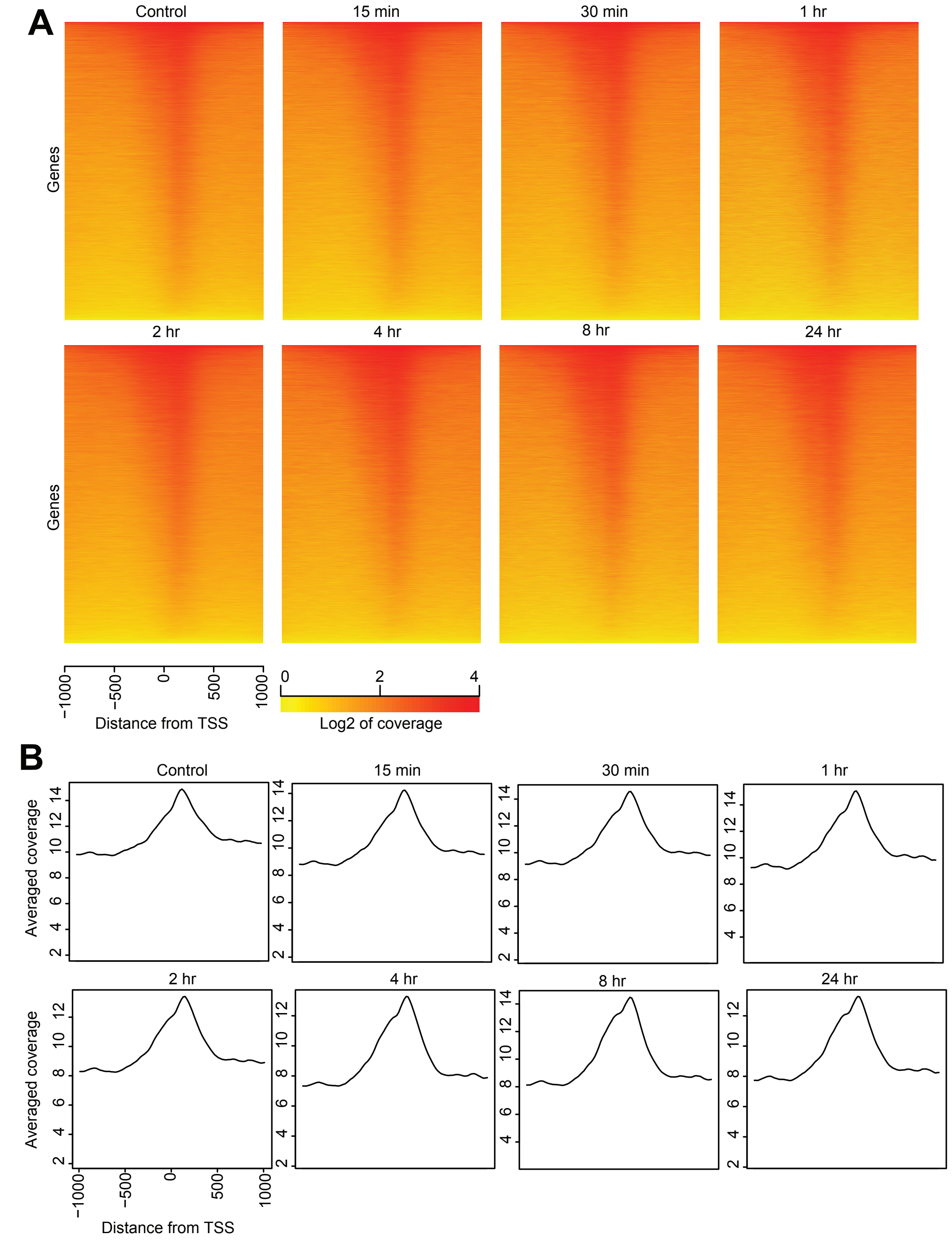
A
Control
15 min
30 min
1 hr
Genes
2 hr
4 hr
8 hr
24 hr
Genes
−1000
−500
0
500
1000
Distance from TSS
0
2
4
Log2 of coverage
B
Control
15 min
30 min
1 hr
Averaged coverage
2 hr
4 hr
8 hr
24 hr
Averaged coverage
Distance from TSS

Figure S5. ATAC-seq coverage around the Transcription Start Site (TSS) for each time point of our LCO time course dataset. (**A**) Shown are heatmaps prepared with the ChIPpeakAnno library featureAlignedHeatmap function for ±1 kbp regions around annotated TSS sites in the *Medicago* v5 genome. The scale is log2 of counts from the data. (**B**) Line profiles of the same data averaged across all TSSs.

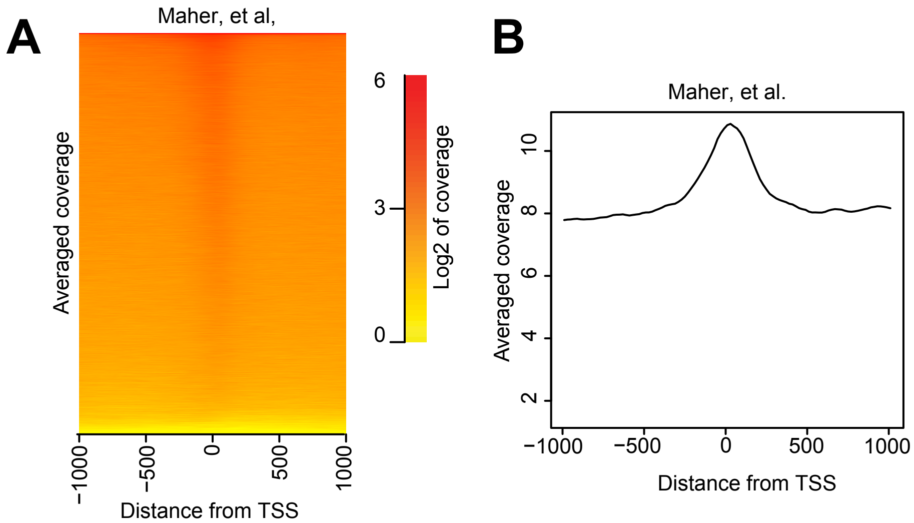

Figure S6. ATAC-seq coverage around the TSS for the Maher et al. Medicago ATAC-seq data. (**A**) Heatmap of TSSs similar to **Figure S5**. (**B**) Line plot of average TSS similar to **Figure S5**. Both are comparable to the analogous results of our ATAC-seq data set (**Figure S5**).

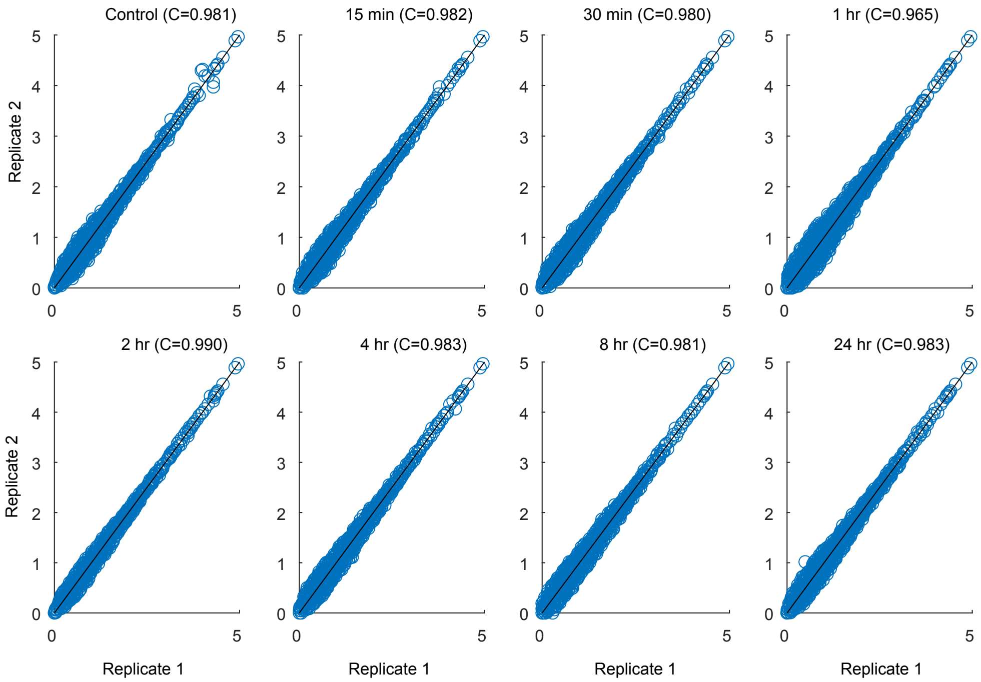

Figure S7. Scatter plots of aggregated promoter signal values of technical replicates of our ATAC-seq data for all time points. The Pearson's correlation values are listed on top of each plot.

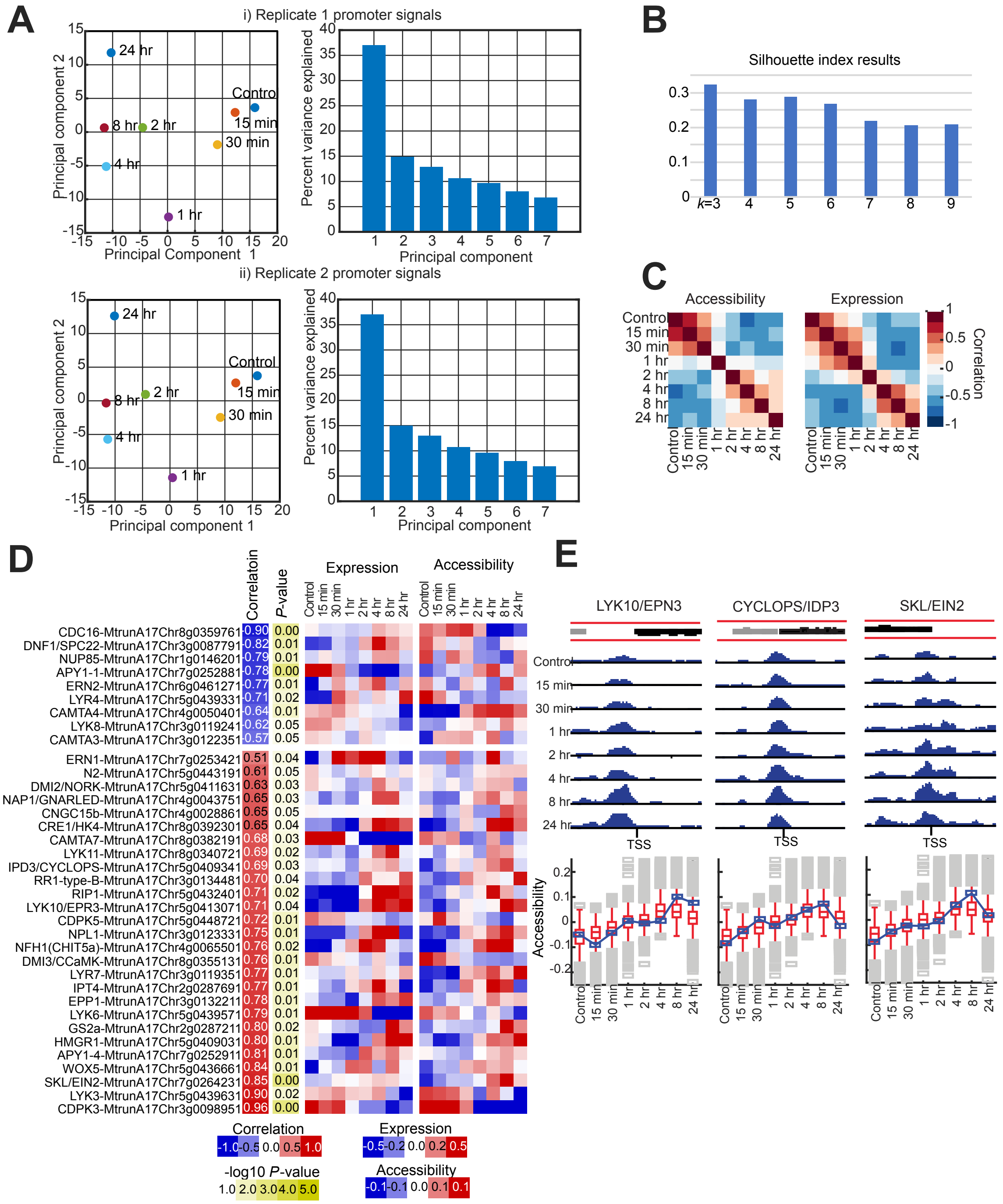
A
i) Replicate 1 promoter signals
24 hr
Control
15 min
8 hr
2 hr
30 min
4 hr
1 hr
Principal component 2
Principal Component 1
Percent variance explained
Principal component
ii) Replicate 2 promoter signals
Principal component 1
B
Silhouette index results
k=3
C
Accessibility
Expression
Correlation
Control
15 min
30 min
1 hr
2 hr
4 hr
8 hr
24 hr
D
Correlatoin
P-value
Expression
Accessibility
CDC16-MtrunA17Chr8g0359761 -0.90 0.00
DNF1/SPC22-MtrunA17Chr3g0087791 -0.82 0.01
NUP85-MtrunA17Chr1g0146201 -0.79 0.01
APY1-1-MtrunA17Chr7g0252881 -0.78 0.00
ERN2-MtrunA17Chr6g0461271 -0.77 0.01
LYR4-MtrunA17Chr5g0439331 -0.71 0.02
CAMTA4-MtrunA17Chr4g0050401 -0.64 0.01
LYK8-MtrunA17Chr3g0119241 -0.62 0.05
CAMTA3-MtrunA17Chr3g0122351 -0.57 0.05
ERN1-MtrunA17Chr7g0253421 0.51 0.04
N2-MtrunA17Chr5g0443191 0.61 0.05
DMI2/NORK-MtrunA17Chr5g0411631 0.63 0.03
NAP1/GNARLED-MtrunA17Chr4g0043751 0.65 0.03
CNGC15b-MtrunA17Chr4g0028861 0.65 0.05
CRE1/HK4-MtrunA17Chr8g0392301 0.65 0.04
CAMTA7-MtrunA17Chr8g0382191 0.68 0.03
LYK11-MtrunA17Chr8g0340721 0.69 0.02
IPD3/CYCLOPS-MtrunA17Chr5g0409341 0.69 0.03
RR1-type-B-MtrunA17Chr3g0134481 0.70 0.04
RIP1-MtrunA17Chr5g0432401 0.71 0.02
LYK10/EPR3-MtrunA17Chr5g0413071 0.71 0.04
CDPK5-MtrunA17Chr5g0448721 0.72 0.01
NPL1-MtrunA17Chr3g0123331 0.75 0.01
NFH1(CHIT5a)-MtrunA17Chr4g0065501 0.76 0.02
DMI3/CCaMK-MtrunA17Chr8g0355131 0.76 0.01
LYR7-MtrunA17Chr3g0119351 0.77 0.01
IPT4-MtrunA17Chr2g0287691 0.77 0.01
EPP1-MtrunA17Chr3g0132211 0.78 0.01
LYK6-MtrunA17Chr5g0439571 0.79 0.01
GS2a-MtrunA17Chr2g0287211 0.80 0.02
HMGR1-MtrunA17Chr5g0409031 0.80 0.01
APY1-4-MtrunA17Chr7g0252911 0.81 0.01
WOX5-MtrunA17Chr5g0436661 0.84 0.01
SKL/EIN2-MtrunA17Chr7g0264231 0.85 0.00
LYK3-MtrunA17Chr5g0439631 0.90 0.02
CDPK3-MtrunA17Chr3g0098951 0.96 0.00
Correlation
-log10 P-value
Expression
Accessibility
E
LYK10/EPN3
CYCLOPS/IDP3
SKL/EIN2
TSS
Accessibility

Figure S8. Gene promoter chromatin accessibility analysis. (A) Principal Components Analysis (PCA) results of aggregated (±2 kbp of TSS) promoter signals for the ATAC-seq technical replicates analyzed separately. PCA shows similar trends as in the combined data (**Figure 3D**). **(B)** Silhouette index (SI) for k-means clustering applied to the zero-meaned promoter accessibility data, motivating a choice of k=6 clusters (after which SI sharply decreases). **(C)** Pearson correlation of the promoter accessibility and gene expression data (with zero-mean transformation) across time points. **(D)** Gene expression and promoter accessibility profiles of 36 nodulation genes of interest with significant positive or negative correlation. Shown are the zero-meaned values. **(E)** Normalized coverage tracks for promoter regions (±2 kbp) for known nodulation genes of interest (prepared in part with Integrative Genomics Viewer (IGV)). In black are shown the genomic coordinates of the genes of interest, and in gray are nearby genes (if present).

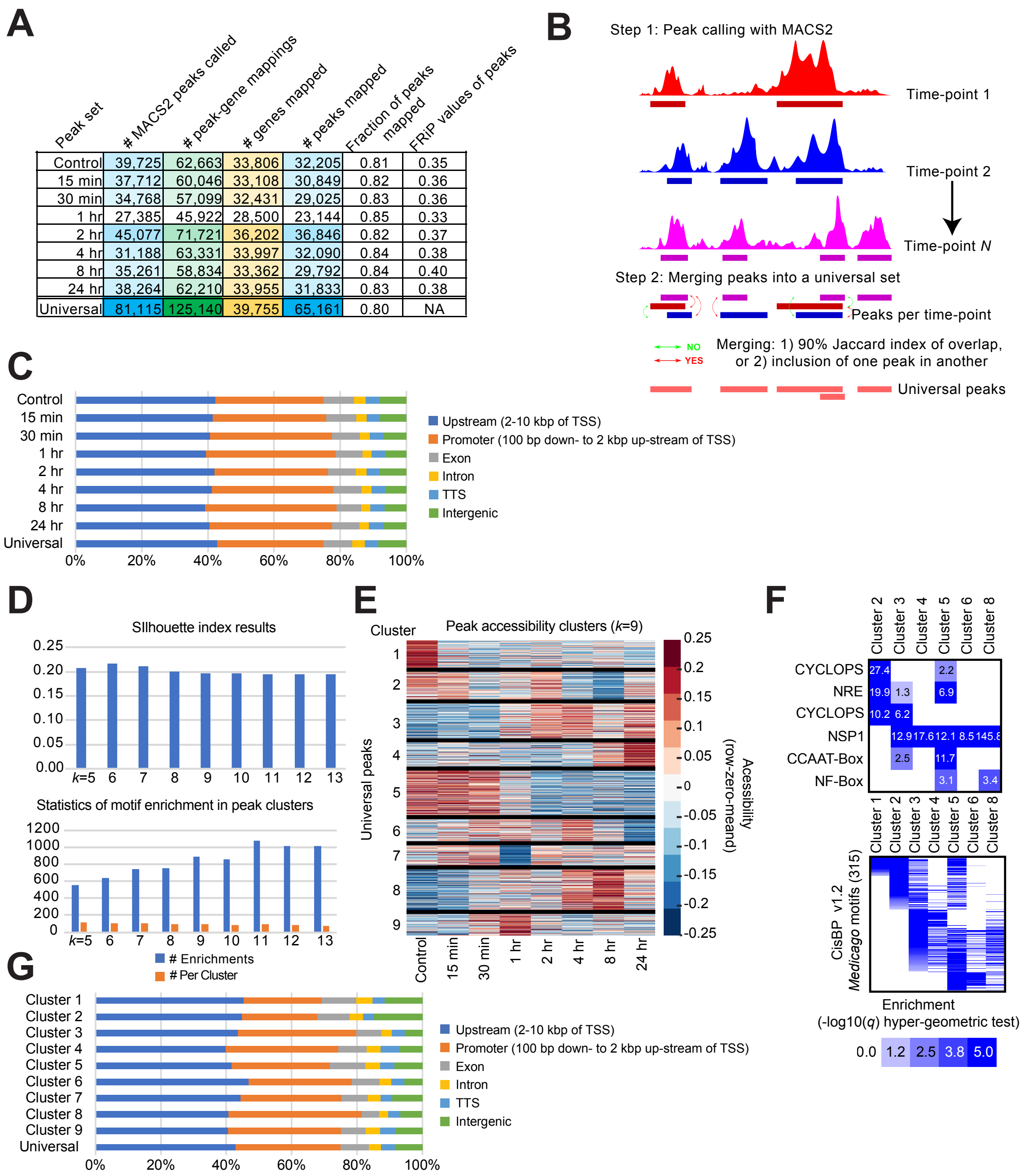

Peak set	# MACS2 peaks called	# peak-gene mappings	# genes mapped	# peaks mapped	Fraction of peaks mapped	FRiP values of peaks
Control	39,725	62,663	33,806	32,205	0.81	0.35
15 min	37,712	60,046	33,108	30,849	0.82	0.36
30 min	34,768	57,099	32,431	29,025	0.83	0.36
1 hr	27,385	45,922	28,500	23,144	0.85	0.33
2 hr	45,077	71,721	36,202	36,846	0.82	0.37
4 hr	31,188	63,331	33,997	32,090	0.84	0.38
8 hr	35,261	58,834	33,362	29,792	0.84	0.40
24 hr	38,264	62,210	33,955	31,833	0.83	0.38
Universal	81,115	125,140	39,755	65,161	0.80	NA

Figure S9. Summary of ATAC-seq peak calling and accessibility analysis. (A) MACS2 peak calling statistics, including number of peaks mapped to genes within 10 kbp upstream to 1 kbp downstream of a gene TSS, fraction of peaks mapped, and fractions of fragments spanning peaks (or Fir values) for each time-point. **(B)** Peak merging procedure to define a universal peak set across time points. Peaks are merged using the criteria of 90% Jaccard distance overlap or one peak being contained in another. **(C)** Genomic annotations of peaks per time point and for the universal set. See **Methods** for details. **(D)** Silhouette index scores and numbers of motif enrichments for k-means clusters of accessibility profiles for the universal peaks. **(E)** Temporal patterns of accessibility for the universal peak regions for k=9 clusters. **(F)** Enrichment (-log(q-value)) for motifs of interest (top) and general CisBP motifs (bottom) for the universal peak accessibility clusters. **(G)** Genomic annotations of peaks by k-means cluster assignment. Clusters 1, 2 and 7 present a higher proportion of peaks annotated to intergenic regions compared to peaks in other clusters (hypergeometric test P<0.05), indicative of distal regulation.

A

Setting (p1=5, and p3=0)	Correlation: Full data set	Correlation: CV training	Correlation: CV test - mean across folds	Correlation: CV test- concatenated across folds	Comparison to ESCAROLE modules: Control	15 min	30 min	1 hr	2 hr	4 hr	8 hr	24 hr	Mean	Silhouette index of modules: Control	15 min	30 min	1 hr	2 hr	4 hr	8 hr	24 hr	Mean
P2=30	0.404	0.446	0.273	0.275	0.85	0.85	0.84	0.84	0.84	0.84	0.84	0.84	0.84	0.532	0.549	0.531	0.483	0.517	0.530	0.491	0.482	0.514
P2=35	0.405	0.447	0.274	0.276	0.85	0.85	0.84	0.83	0.83	0.84	0.84	0.83	0.84	0.531	0.549	0.530	0.483	0.517	0.530	0.489	0.482	0.514
P2=40	0.403	0.446	0.273	0.274	0.85	0.85	0.84	0.84	0.84	0.84	0.84	0.84	0.84	0.532	0.550	0.531	0.483	0.518	0.530	0.490	0.483	0.515
P2=45	0.404	0.447	0.275	0.276	0.85	0.85	0.84	0.83	0.84	0.84	0.84	0.84	0.84	0.532	0.549	0.531	0.482	0.518	0.529	0.489	0.483	0.514
P2=50	0.404	0.446	0.275	0.276	0.85	0.85	0.84	0.84	0.84	0.84	0.84	0.83	0.84	0.532	0.548	0.531	0.483	0.518	0.530	0.489	0.483	0.514
P2=55	0.404	0.445	0.273	0.275	0.85	0.85	0.84	0.84	0.84	0.84	0.84	0.84	0.84	0.531	0.549	0.531	0.483	0.517	0.530	0.491	0.482	0.514
P2=60	0.405	0.447	0.274	0.276	0.85	0.85	0.84	0.84	0.83	0.84	0.83	0.83	0.84	0.531	0.548	0.531	0.482	0.517	0.530	0.489	0.482	0.514
P2=75	0.404	0.446	0.274	0.276	0.85	0.85	0.84	0.83	0.83	0.84	0.84	0.83	0.84	0.532	0.548	0.531	0.482	0.517	0.530	0.489	0.481	0.514
P2=100	0.403	0.446	0.273	0.275	0.85	0.85	0.84	0.84	0.83	0.84	0.84	0.83	0.84	0.531	0.549	0.531	0.482	0.517	0.530	0.489	0.483	0.514

B

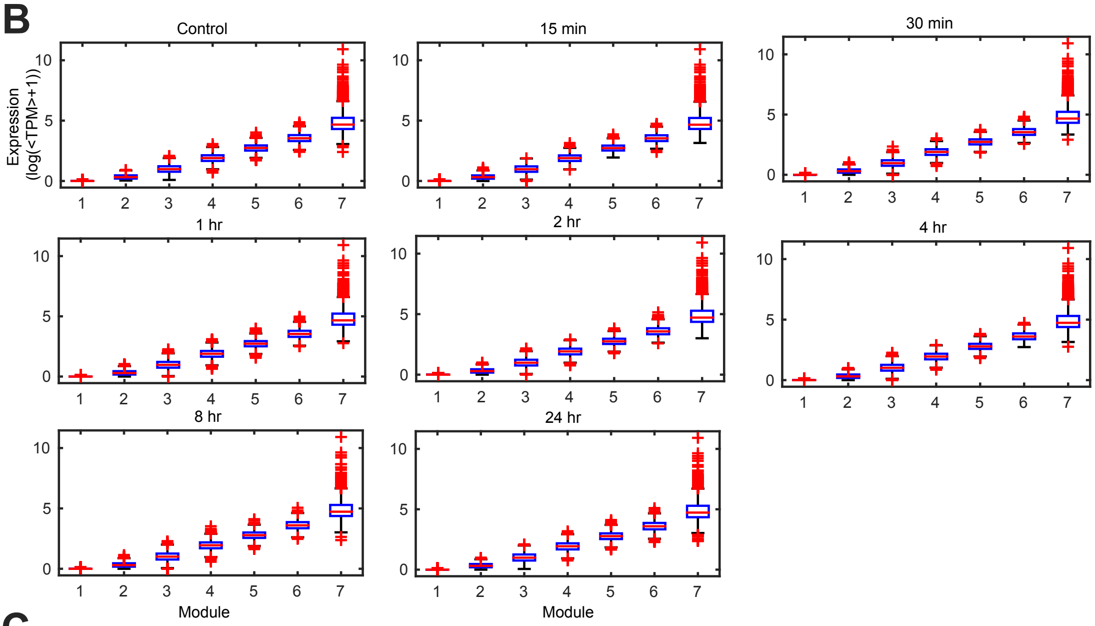

C

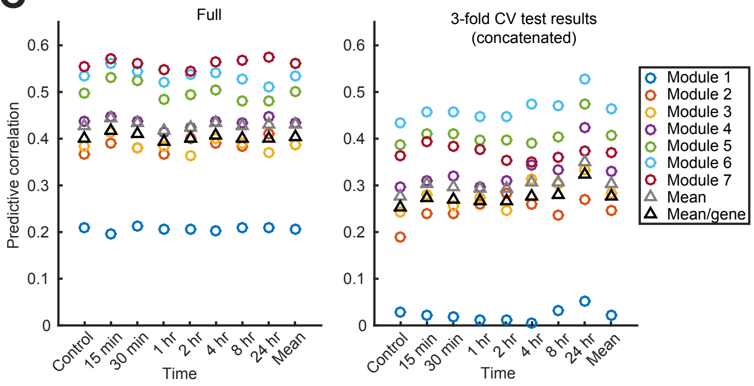

Figure S10. DRMN hyper-parameter tuning. (A) Shown in blue is mean correlation of observed and predicted expression (averaged across modules) for tuning the fused-lasso hyper parameter p_2=30-60, given p_1=5 and p_3=0. The selected hyper-parameter setting, p_1=5, p_2=45, p_3=0 have the highest correlation and predictive power, justifying their selection for DRMN application. The middle set of columns are the mean similarity of ESCAROLE and DRMN modules based on F-score. The rightmost set of columns are DRMN module silhouette index. **(B)** Expression levels across DRMN modules (x-axis labels) for all timepoints. The box plots show the significance of difference in expression across the modules, and similarity of expression levels of the same module across time points. **(C)** Pearson's correlation of observed and predicted expression for the full data set (left) and for three-fold cross validation results (right). The average correlation across modules (gray triangle) and weighted average (weight corresponding to gene set size, black triangle) summarize the correlations at each time point and averaged across all time points (right-most column of points in both panels).

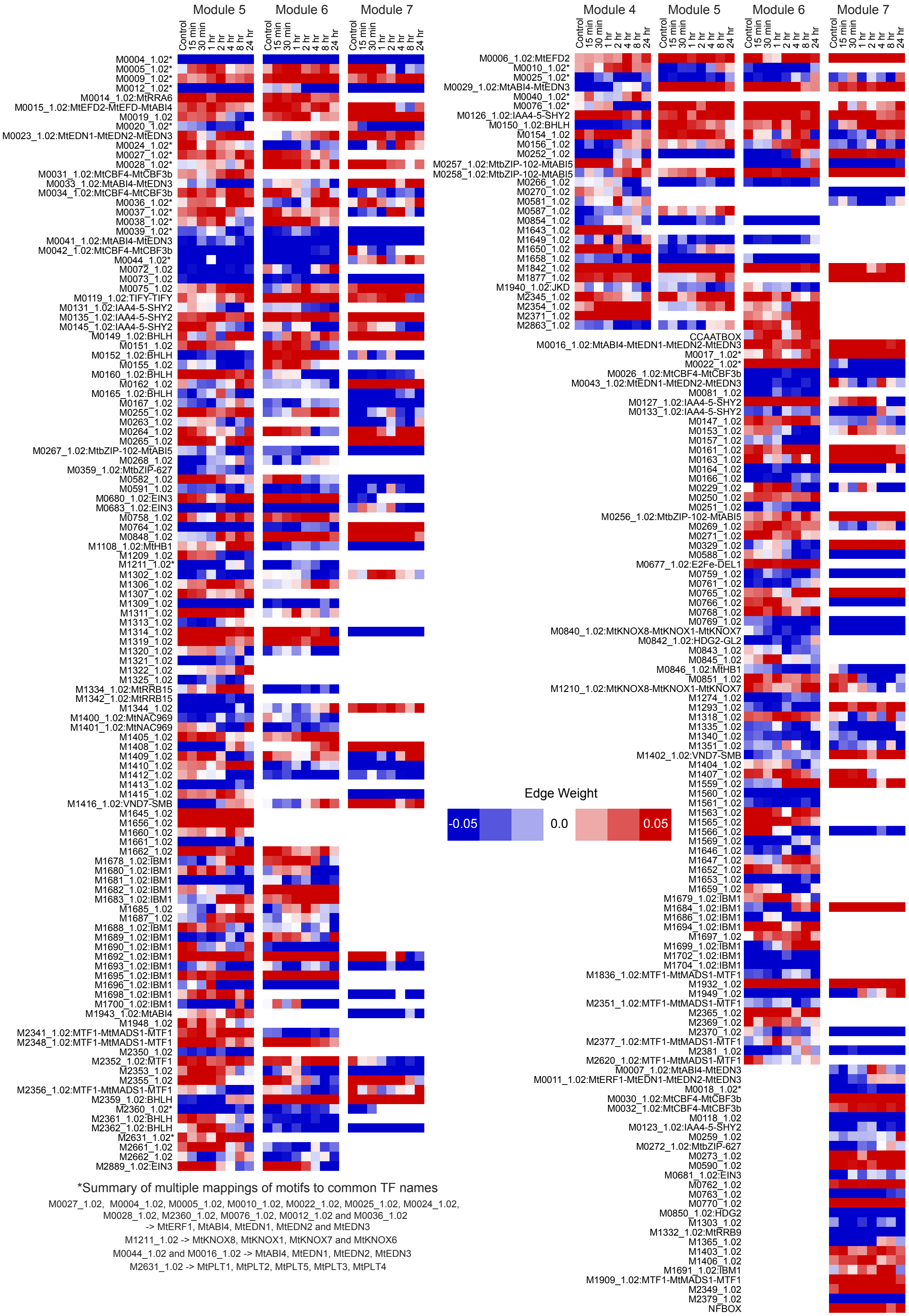

*Summary of multiple mappings of motifs to common TF names

M0027_1.02, M0004_1.02, M0005_1.02, M0010_1.02, M0022_1.02, M0025_1.02, M0024_1.02,
M0028_1.02, M2360_1.02, M0076_1.02, M0012_1.02 and M0036_1.02
-> MtERF1, MtABI4, MtEDN1, MtEDN2 and MtEDN3
M1211_1.02 -> MtKNOX8, MtKNOX1, MtKNOX7 and MtKNOX6
M0044_1.02 and M0016_1.02 -> MtABI4, MtEDN1, MtEDN2, MtEDN3
M2631_1.02 -> MtPLT1, MtPLT2, MtPLT5, MtPLT3, MtPLT4

Figure S11. DRMN edge weights for each module. Shown are the inferred regression weights for each module and regulator association in the DRMN analysis. Only module-regulator edges with an absolute value >0.02 in any time point and supported with enrichment (FDR-corrected hypergeometric test $q<0.05$) for the corresponding motif in the respective module are shown. Edges meeting these criteria were found only for modules 4-7.

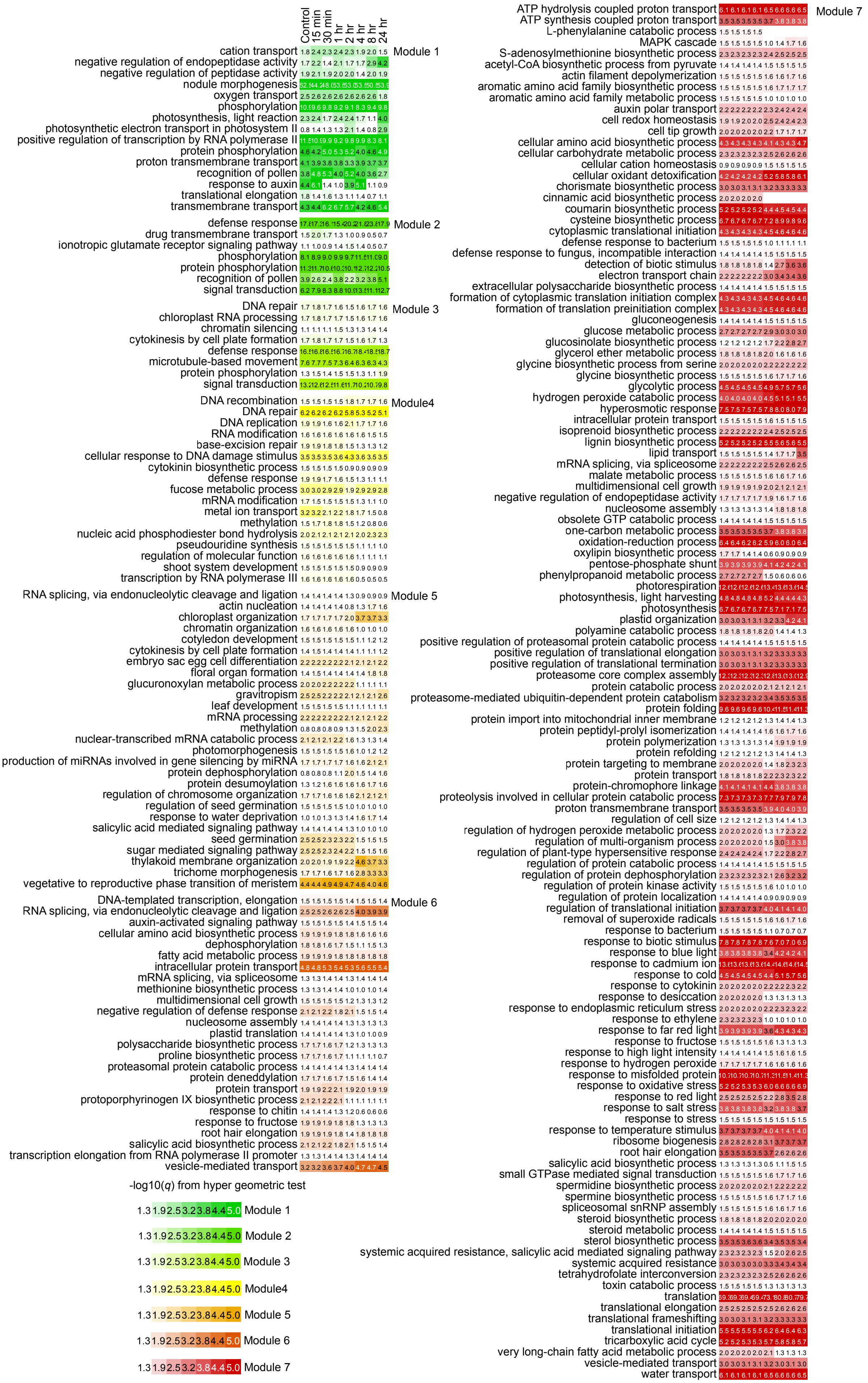
Control
15 min
30 min
1 hr
2 hr
4 hr
8 hr
24 hr
Module 1
cation transport
negative regulation of endopeptidase activity
negative regulation of peptidase activity
nodule morphogenesis
oxygen transport
phosphorylation
photosynthesis, light reaction
photosynthetic electron transport in photosystem II
positive regulation of transcription by RNA polymerase II
protein phosphorylation
proton transmembrane transport
recognition of pollen
response to auxin
translational elongation
transmembrane transport
Module 2
defense response
drug transmembrane transport
ionotropic glutamate receptor signaling pathway
phosphorylation
protein phosphorylation
recognition of pollen
signal transduction
Module 3
DNA repair
chloroplast RNA processing
chromatin silencing
cytokinesis by cell plate formation
defense response
microtubule-based movement
protein phosphorylation
signal transduction
Module4
DNA recombination
DNA repair
DNA replication
RNA modification
base-excision repair
cellular response to DNA damage stimulus
cytokinin biosynthetic process
defense response
fucose metabolic process
mRNA modification
metal ion transport
methylation
nucleic acid phosphodiester bond hydrolysis
pseudouridine synthesis
regulation of molecular function
shoot system development
transcription by RNA polymerase III
Module 5
RNA splicing, via endonucleolytic cleavage and ligation
actin nucleation
chloroplast organization
chromatin organization
cotyledon development
cytokinesis by cell plate formation
embryo sac egg cell differentiation
floral organ formation
glucuronoxylan metabolic process
gravitropism
leaf development
mRNA processing
methylation
nuclear-transcribed mRNA catabolic process
photomorphogenesis
production of miRNAs involved in gene silencing by miRNA
protein dephosphorylation
protein desumoylation
regulation of chromosome organization
regulation of seed germination
response to water deprivation
salicylic acid mediated signaling pathway
seed germination
sugar mediated signaling pathway
thylakoid membrane organization
trichome morphogenesis
vegetative to reproductive phase transition of meristem
Module 6
DNA-templated transcription, elongation
RNA splicing, via endonucleolytic cleavage and ligation
auxin-activated signaling pathway
cellular amino acid biosynthetic process
dephosphorylation
fatty acid metabolic process
intracellular protein transport
mRNA splicing, via spliceosome
methionine biosynthetic process
multidimensional cell growth
negative regulation of defense response
nucleosome assembly
plastid translation
polysaccharide biosynthetic process
proline biosynthetic process
proteasomal protein catabolic process
protein deneddylation
protein transport
protoporphyrinogen IX biosynthetic process
response to chitin
response to fructose
root hair elongation
salicylic acid biosynthetic process
transcription elongation from RNA polymerase II promoter
vesicle-mediated transport
-log10(q) from hyper geometric test
1.3 1.9 2.5 3.2 3.8 4.4 5.0 Module 1
1.3 1.9 2.5 3.2 3.8 4.4 5.0 Module 2
1.3 1.9 2.5 3.2 3.8 4.4 5.0 Module 3
1.3 1.9 2.5 3.2 3.8 4.4 5.0 Module4
1.3 1.9 2.5 3.2 3.8 4.4 5.0 Module 5
1.3 1.9 2.5 3.2 3.8 4.4 5.0 Module 6
1.3 1.9 2.5 3.2 3.8 4.4 5.0 Module 7
Module 7
ATP hydrolysis coupled proton transport
ATP synthesis coupled proton transport
L-phenylalanine catabolic process
MAPK cascade
S-adenosylmethionine biosynthetic process
acetyl-CoA biosynthetic process from pyruvate
actin filament depolymerization
aromatic amino acid family biosynthetic process
aromatic amino acid family metabolic process
auxin polar transport
cell redox homeostasis
cell tip growth
cellular amino acid biosynthetic process
cellular carbohydrate metabolic process
cellular cation homeostasis
cellular oxidant detoxification
chorismate biosynthetic process
cinnamic acid biosynthetic process
coumarin biosynthetic process
cysteine biosynthetic process
cytoplasmic translational initiation
defense response to bacterium
defense response to fungus, incompatible interaction
detection of biotic stimulus
electron transport chain
extracellular polysaccharide biosynthetic process
formation of cytoplasmic translation initiation complex
formation of translation preinitiation complex
gluconeogenesis
glucose metabolic process
glucosinolate biosynthetic process
glycerol ether metabolic process
glycine biosynthetic process from serine
glycine biosynthetic process
glycolytic process
hydrogen peroxide catabolic process
hyperosmotic response
intracellular protein transport
isoprenoid biosynthetic process
lignin biosynthetic process
lipid transport
mRNA splicing, via spliceosome
malate metabolic process
multidimensional cell growth
negative regulation of endopeptidase activity
nucleosome assembly
obsolete GTP catabolic process
one-carbon metabolic process
oxidation-reduction process
oxylipin biosynthetic process
pentose-phosphate shunt
phenylpropanoid metabolic process
photorespiration
photosynthesis, light harvesting
photosynthesis
plastid organization
polyamine catabolic process
positive regulation of proteasomal protein catabolic process
positive regulation of translational elongation
positive regulation of translational termination
proteasome core complex assembly
protein catabolic process
proteasome-mediated ubiquitin-dependent protein catabolism
protein folding
protein import into mitochondrial inner membrane
protein peptidyl-prolyl isomerization
protein polymerization
protein refolding
protein targeting to membrane
protein transport
protein-chromophore linkage
proteolysis involved in cellular protein catabolic process
proton transmembrane transport
regulation of cell size
regulation of hydrogen peroxide metabolic process
regulation of multi-organism process
regulation of plant-type hypersensitive response
regulation of protein catabolic process
regulation of protein dephosphorylation
regulation of protein kinase activity
regulation of protein localization
regulation of translational initiation
removal of superoxide radicals
response to bacterium
response to biotic stimulus
response to blue light
response to cadmium ion
response to cold
response to cytokinin
response to desiccation
response to endoplasmic reticulum stress
response to ethylene
response to far red light
response to fructose
response to high light intensity
response to hydrogen peroxide
response to misfolded protein
response to oxidative stress
response to red light
response to salt stress
response to stress
response to temperature stimulus
ribosome biogenesis
root hair elongation
salicylic acid biosynthetic process
small GTPase mediated signal transduction
spermidine biosynthetic process
spermine biosynthetic process
spliceosomal snRNP assembly
steroid biosynthetic process
steroid metabolic process
sterol biosynthetic process
systemic acquired resistance, salicylic acid mediated signaling pathway
systemic acquired resistance
tetrahydrofolate interconversion
toxin catabolic process
translation
translational elongation
translational frameshifting
translational initiation
tricarboxylic acid cycle
very long-chain fatty acid metabolic process
vesicle-mediated transport
water transport

Figure S12. Gene ontology enrichments for DRMN modules. Each color represents a different module. The color map represents the -log10(*q*-value) for the significance of enrichment, where the *q*-value is the FDR corrected *P*-value from a hypergeometric test for overlap.

A

Threshold: No. missmatches	ESCAROLE				DRMN			
	No. genes clustered	No. clusters	No. GO enrichments	No. clusters with GO enrichments	No. genes clustered	No. clusters	No. GO enrichments	No. clusters with GO enrichments
0	9,771	228	72	33	8,837	163	414	74
1	10,993	198	87	33	9,633	133	462	64
2	11,612	112	80	31	10,176	79	505	51
3	11,932	71	59	26	10,409	55	505	39

B

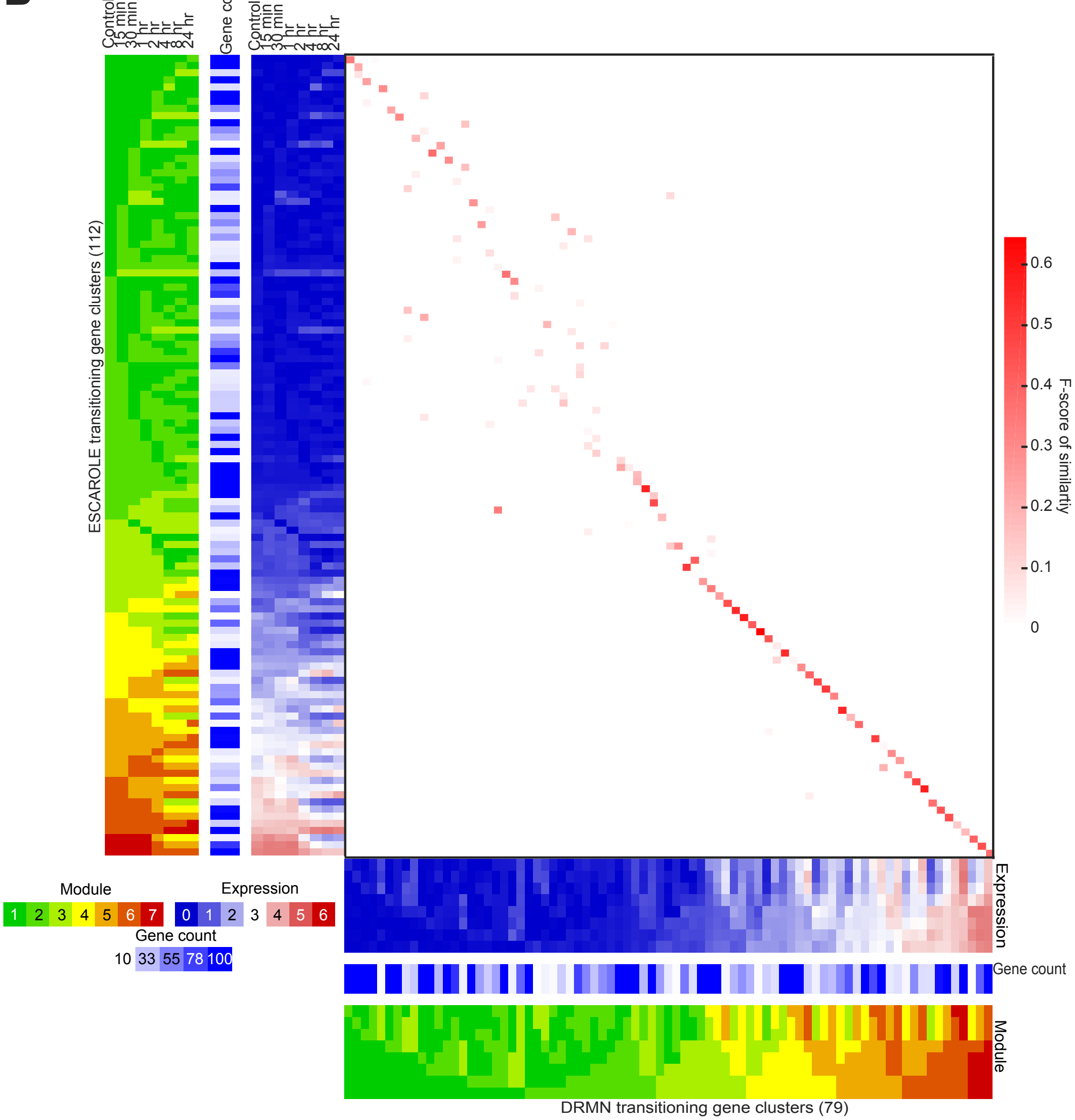

Figure S13. Comparison of ESCAROLE and DRMN transitioning gene sets. (A) Clustering statistics for ESCAROLE and DRMN transitioning gene sets. Shown are the numbers of gene ontology (GO) enrichments and numbers of clusters with such enrichments versus the threshold for cutting the dendrogram of hierarchical clustering (row). The thresholds represent 0-3 mismatches in module assignment across the 8-point time course (see **Methods**). The results presented in this work corresponds to 2 mismatches (purple) which maximizes number of genes included, and a near-maximal number of clusters with enrichments. **(B)** Comparison of individual ESCAROLE and DRMN transitioning gene sets for gene membership. Along the vertical and horizontal axes are the mean module assignment, cluster size, and mean gene expression profiles (see also **Figures 2B** and **5D)**, respectively. In the center, F-scores of overlaps between individual gene sets are presented (white-red color scale). The gene sets are 77% similar in gene content, but 26% similar on average for each pair of sets.

Cluster	Size	λ Setting	Correlation									Number of regulators									Fraction								
			λ=0.20	λ=0.30	λ=0.40	λ=0.50	λ=0.60	λ=0.70	λ=0.20	λ=0.90	λ=0.99	λ=0.20	λ=0.30	λ=0.40	λ=0.50	λ=0.60	λ=0.70	λ=0.20	λ=0.90	λ=0.99	λ=0.20	λ=0.30	λ=0.40	λ=0.50	λ=0.60	λ=0.70	λ=0.20	λ=0.90	λ=0.99
161	10		0.05	-0.05	-0.12	-0.20	-0.24	-0.26	-0.24	-0.10	-0.07																		
232	10	0.80	0.33	0.33	0.33	0.33	0.33	0.24	0.19	0.17	0.19	9	6	3	2	2	2	1	1	1	0.900	0.600	0.300	0.200	0.200	0.200	0.100	0.100	0.100
158	11		0.33	0.23	0.16	0.13	0.10	0.13	0.17	0.12	0.08																		
173	11		0.26	0.17	0.12	0.10	0.09	0.05	-0.03	-0.08	-0.07		11									1.000							
185	12	0.90	0.48	0.42	0.39	0.40	0.40	0.46	0.53	0.57	0.43		11	8	6	3	2	2	1	1		0.917	0.667	0.500	0.250	0.167	0.167	0.083	0.083
205	12		0.28	0.19	0.11	0.04	-0.04	-0.13	-0.17	-0.16	-0.14																		
140	13		0.41	0.17	0.12	0.10	0.08	0.26	0.29	0.35	0.36																		
184	13	0.80	0.44	0.41	0.37	0.34	0.30	0.25	0.16	0.12	0.14	13	7	7	4	3	2	1			1.000	0.538	0.538	0.308	0.231	0.154	0.077		
212	13		0.33	0.26	0.20	0.15	0.11	0.06	0.08	0.09	0.09																		
114	14		0.13	0.06	0.04	0.07	0.12	0.13	0.15	0.16	0.16																		
156	14		0.23	0.15	0.12	0.08	0.05	0.05	0.08	0.08	0.08																		
109	15		0.15	-0.01	-0.07	-0.11	-0.13	-0.14	-0.12	-0.12	-0.19																		
207	15		0.18	0.12	0.09	0.09	0.09	0.07	0.08	0.10	0.11									1									0.067
123	18	0.90	0.55	0.48	0.43	0.38	0.35	0.30	0.27	0.21	0.10			11	9	7	6	2	1				0.611	0.500	0.389	0.333	0.111	0.056	
209	18		0.21	0.15	0.14	0.14	0.12	0.08	0.07	0.07	0.06								1									0.056	
188	21		0.06	0.03	0.01	-0.01	-0.04	-0.08	-0.09	-0.06	-0.06	12	8	8	6	4	4	5	2	1	0.571	0.381	0.381	0.286	0.190	0.190	0.238	0.095	0.048
211	21		0.26	0.11	0.06	0.04	0.02	0.02	0.06	0.09	0.12																		
252	21	0.70	0.64	0.57	0.52	0.47	0.40	0.32	0.21	0.09	0.09	18	13	11	9	8	6				0.857	0.619	0.524	0.429	0.381	0.286			
196	22	0.80	0.30	0.26	0.25	0.25	0.25	0.24	0.24	0.25	0.14					3	3	1	1						0.136	0.136	0.045	0.045	
203	22		0.26	0.15	0.11	0.11	0.16	0.12	0.07	0.10	0.10																		
231	22		0.18	0.09	0.06	0.08	0.12	0.16	0.20	0.20	0.21																		
219	23		0.46	0.36	0.29	0.24	0.22	0.22	0.21	0.18	0.14																		
71	26		0.32	0.27	0.23	0.19	0.21	0.18	0.18	0.19	0.19								1									0.038	
206	26	0.80	0.46	0.41	0.37	0.36	0.34	0.33	0.32	0.31	0.31	14	10	5	3	2	2	1	1	1	0.538	0.385	0.192	0.115	0.077	0.077	0.038	0.038	0.038
200	27	0.80	0.24	0.18	0.16	0.15	0.12	0.09	0.05	0.01	-0.00		16	10	8	6	4	3				0.593	0.370	0.296	0.222	0.148	0.111		
129	28		0.52	0.41	0.34	0.30	0.24	0.19	0.14	0.08	0.06	24									0.857								
155	28		0.11	0.01	-0.02	-0.03	0.02	0.07	0.10	0.14	0.12																		
191	29	0.80	0.32	0.22	0.18	0.16	0.13	0.11	0.12	0.14	0.14							1	1	1							0.034	0.034	0.034
75	31		0.25	0.15	0.10	0.06	0.04	0.02	-0.02	-0.03	-0.05																		
107	31	0.90	0.43	0.37	0.35	0.31	0.27	0.25	0.21	0.14	0.12	23	15	14	11	6	4	3	1		0.742	0.484	0.452	0.355	0.194	0.129	0.097	0.032	
163	35		0.26	0.18	0.11	0.08	0.09	0.09	0.09	0.09	0.09									1									0.029
168	35		0.35	0.15	0.04	-0.04	-0.12	-0.17	-0.18	-0.15	-0.11																		
124	36		0.38	0.28	0.22	0.19	0.16	0.17	0.16	0.16	0.16																		
193	37		0.38	0.28	0.21	0.15	0.10	0.06	0.04	0.00	-0.04																		
202	37		0.38	0.25	0.18	0.16	0.17	0.17	0.18	0.18	0.12																		
128	43		0.41	0.29	0.19	0.09	0.03	0.03	0.08	0.09	0.05																		
190	43	0.60	0.38	0.30	0.27	0.23	0.20	0.16	0.12	0.04	0.02			16	13	6	6						0.372	0.302	0.140	0.140			
199	44		0.37	0.34	0.32	0.28	0.21	0.11	0.05	0.02	-0.01																		
174	48	0.70	0.38	0.31	0.27	0.22	0.19	0.19	0.16	0.16	0.16		19	15	7	5	2					0.396	0.312	0.146	0.104	0.042			
176	50		0.34	0.21	0.15	0.13	0.12	0.16	0.19	0.19	0.19								1	1								0.020	0.020
108	51	0.70	0.38	0.31	0.25	0.21	0.17	0.16	0.16	0.17	0.17						2	1	1	1						0.039	0.020	0.020	0.020
110	52		0.34	0.25	0.21	0.21	0.23	0.25	0.22	0.15	0.08																		
251	53		0.41	0.34	0.29	0.25	0.20	0.17	0.11	0.12	0.14																		
214	57	0.80	0.37	0.26	0.19	0.15	0.13	0.11	0.10	0.07	0.05	33	24	20	16	15	8	4	1		0.579	0.421	0.351	0.281	0.263	0.140	0.070	0.018	
210	59	0.50	0.56	0.44	0.34	0.27	0.20	0.13	0.07	0.02	-0.05	32	28	25	19						0.542	0.475	0.424	0.322					
169	63		0.37	0.26	0.20	0.18	0.17	0.15	0.16	0.16	0.16								1	1								0.016	0.016
112	67	0.80	0.29	0.25	0.24	0.23	0.21	0.20	0.16	0.12	0.07	30	23	16	10	8	7	4	2		0.448	0.343	0.239	0.149	0.119	0.104	0.060	0.030	
183	70	0.60	0.30	0.26	0.24	0.22	0.18	0.16	0.14	0.11	0.07	32	18	14	8	3	2	2	2		0.457	0.257	0.200	0.114	0.043	0.029	0.029	0.029	
208	74	0.70	0.40	0.35	0.29	0.24	0.21	0.15	0.11	0.10	0.09						6	2	2	2						0.081	0.027	0.027	0.027
218	77		0.32	0.18	0.12	0.09	0.06	0.02	-0.04	-0.09	-0.04																		
162	81	0.40	0.47	0.37	0.30	0.23	0.15	0.10	0.07	0.09	0.09	38	30	21							0.469	0.370	0.259						
127	94	0.70	0.38	0.33	0.29	0.26	0.23	0.19	0.17	0.13	0.12	33	29	23	19	10	5	3			0.351	0.309	0.245	0.202	0.106	0.053	0.032		
126	118		0.40	0.31	0.26	0.21	0.18	0.15	0.11	0.11	0.12									1									0.008
249	119	0.70	0.41	0.30	0.23	0.19	0.15	0.12	0.08	0.05	0.07				20	13	8							0.168	0.109	0.067			
248	123	0.60	0.51	0.47	0.45	0.44	0.42	0.40	0.38	0.35	0.30	35	25	18	13	7	4	2	2		0.285	0.203	0.146	0.106	0.057	0.033	0.016	0.016	
165	131		0.47	0.40	0.35	0.30	0.25	0.21	0.16	0.09	0.06			28	25								0.214	0.191					
157	142		0.28	0.20	0.16	0.15	0.15	0.14	0.13	0.14	0.15																		
181	158		0.42	0.29	0.22	0.18	0.15	0.13	0.09	0.06	0.03																		
204	190		0.34	0.27	0.26	0.26	0.25	0.22	0.18	0.18	0.18									1									0.005
253	214		0.38	0.25	0.17	0.11	0.06	0.04	0.02	0.03	0.02																		
217	216		0.33	0.22	0.15	0.11	0.08	0.05	0.02	0.02	0.01																		
197	246		0.36	0.24	0.16	0.09	0.03	-0.01	-0.03	-0.02	-0.03																		
220	251	0.30	0.37	0.30	0.24	0.19	0.18	0.17	0.15	0.15	0.14	23	15	10	3	1	1				0.092	0.060	0.040	0.012	0.004	0.004			
213	266		0.36	0.26	0.20	0.17	0.15	0.11	0.07	0.03	0.01																		
189	289		0.33	0.22	0.15	0.10	0.07	0.03	0.01	-0.02	-0.03																		
106	292	0.40	0.42	0.38	0.36	0.33	0.31	0.30	0.27	0.19	0.15	35	24	13	7	4	4	2	2	1	0.120	0.082	0.045	0.024	0.014	0.014	0.007	0.007	0.003
215	299		0.36	0.23	0.16	0.12	0.10	0.09	0.09	0.10	0.11								1	1								0.003	0.003
175	303		0.32	0.21	0.15	0.10	0.06	0.04	0.05	0.07	0.09																		
177	306		0.35	0.24	0.18	0.13	0.10	0.08	0.09	0.09	0.09																		
125	338		0.52	0.38	0.29	0.22	0.17	0.13	0.09	0.05	0.06									1									0.003
256	358	0.60	0.44	0.35	0.29	0.25	0.21	0.18	0.14	0.10	0.06	44	40	32	25	20	10	4	2		0.123	0.112	0.089	0.070	0.056	0.028	0.011	0.006	
250	381	0.50	0.45	0.39	0.35	0.32	0.30	0.28	0.22	0.14	0.13	42	35	27	19	14	9	4	1	1	0.110	0.092	0.071	0.050	0.037	0.024	0.010	0.003	0.003
254	417	0.50	0.42	0.35	0.31	0.28	0.26	0.24	0.22	0.18	0.13	41	41	33	26	19	12	5	4		0.098	0.098	0.079	0.062	0.046	0.029	0.012	0.010	
182	447	0.40	0.42	0.32	0.26	0.22	0.19	0.17	0.15	0.12	0.07	53	36	25	20	14	11	8	4	1	0.119	0.081	0.056	0.045	0.031	0.025	0.018	0.009	0.002
201	497	0.40	0.46	0.39	0.35	0.31	0.28	0.25	0.23	0.19	0.12	41	38	30	34	24	14	9	5		0.082	0.076	0.060	0.068	0.048	0.028	0.018	0.010	
186	529	0.50	0.44	0.34	0.29	0.25	0.22	0.18	0.13	0.11	0.11				23	14	6	2	1	1				0.043	0.026	0.011	0.004	0.002	0.002
159	550	0.40	0.43	0.35	0.30	0.27	0.24	0.22	0.19	0.14	0.07	40	42	27	23	14	12	7	2	1	0.073	0.076	0.049	0.042	0.025	0.022	0.013	0.004	0.002
216	579	0.40	0.43	0.36	0.31	0.29	0.27	0.25	0.22	0.19	0.12	47	43	28	24	18	14	6	3		0.081	0.074	0.048	0.041	0.031	0.024	0.010	0.005	
255	598	0.30	0.46	0.40	0.36	0.33	0.30	0.27	0.22	0.16	0.16	49	39	26	17	9	4	4	1	1	0.082	0.065	0.043	0.028	0.015	0.007	0.007	0.002	0.002

Cluster size: 10 25 40 55 70 85 100

Setting for λ: 0.0 0.2 0.3 0.5 0.7 0.8 1.0

Correlation: -0.5 -0.3 -0.2 0.0 0.2 0.3 0.5

Number of regulators: 0.0 8.3 16.7 25.0 33.3 41.7 50.0

Fraction: 0.0 0.02 0.0

Figure S14. Hyper-parameter (λ) tuning for MTG-LASSO. The correlation between observed/true and predicted expression using the inferred MTG-LASSO regulator set (red heatmap), the number of significant regulators (blue heatmap center) and the ratio of significant regulators to the numbers of target genes (green) were used to choose an appropriate setting of λ for each transitioning gene cluster, indicated in the second column (Setting for λ).

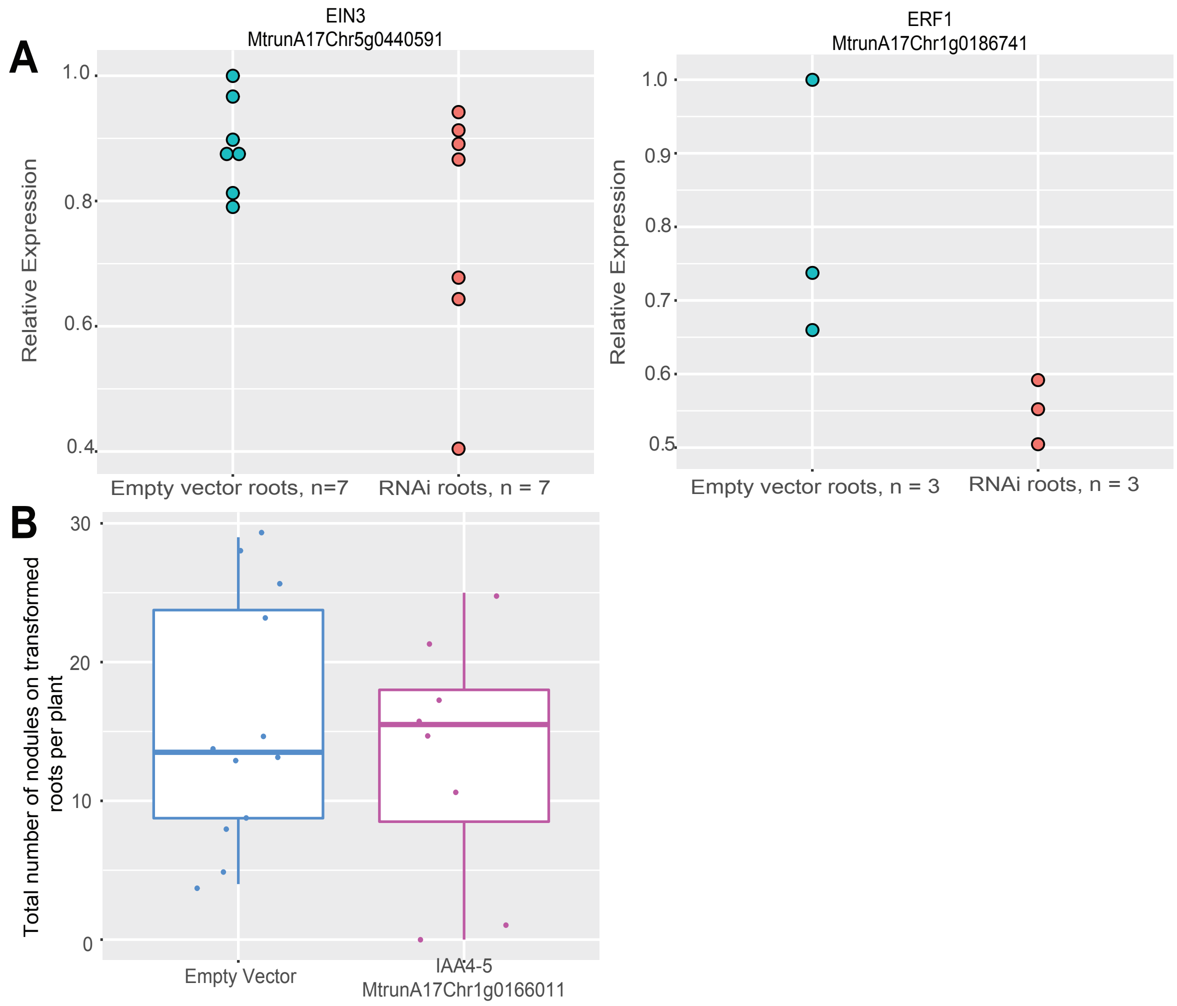

Figure S15. Validation of DRMN regulators with RNAi. (**A**) Expression levels of MtrunA175g0440591 (EIN3) and MtrunA17Chr1g0185741 (ERF1) under RNAi knockdown with an empty vector or the respective RNAi constructs (n=7, and 3 replicates, respectively). (**B**) Knockdown of *MtrunA17Chr1g0166011* (IAA4-5) did not affect the number of nodules formed on composite *M. truncatula* plants (n=20 and 8 replicates for EV control and IAA4-5 knock-down, respectively). Radicles of Jemalong A17 were inoculated (MPMI) [63] with Agrobacterium rhizo-genes MSU440, expressing the RNAi construct, or the empty vector. Three weeks after transformation, plants with transformed roots were transferred to growth pouches and inoculated with *S. meliloti* 1021 harboring pXLGD4 constitutively expressing lacZ. Two weeks after inoculation, live seedlings were stained with X-gal, and the nodules were scores and imaged.
